# Development of a multipurpose scaffold for the display of peptide loops

**DOI:** 10.1093/protein/gzx017

**Published:** 2017-04-24

**Authors:** Maxim Rossmann, Sandra J. Greive, Tommaso Moschetti, Michael Dinan, Marko Hyvönen

**Affiliations:** 2 Department of Biochemistry, University of Cambridge, 80 Tennis Court Road, CambridgeCB2 1GA, UK; 1 Present address: York Structural Biology Laboratory, Department of Chemistry, University of York, Heslington, York YO10 5DD, UK

**Keywords:** Aurora A, CK2alpha, peptide display, protein–protein interactions, thermostable RadA

## Abstract

Protein–protein interactions (PPIs) determine a wide range of biological processes and analysis of these dynamic networks is increasingly becoming a mandatory tool for studying protein function. Using the globular ATPase domain of recombinase RadA as a scaffold, we have developed a peptide display system (RAD display), which allows for the presentation of target peptides, protein domains or full-length proteins and their rapid recombinant production in bacteria. The design of the RAD display system includes differently tagged versions of the scaffold, which allows for flexibility in the protein purification method, and chemical coupling for small molecule labeling or surface immobilization. When combined with the significant thermal stability of the RadA protein, these features create a versatile multipurpose scaffold system. Using various orthogonal biophysical techniques, we show that peptides displayed on the scaffold bind to their natural targets in a fashion similar to linear parent peptides. We use the examples of CK2β/CK2α kinase and TPX2/Aurora A kinase protein complexes to demonstrate that the peptide displayed by the RAD scaffold can be used in PPI studies with the same binding efficacy but at lower costs compared with their linear synthetic counterparts.

## Introduction

Biochemical and biophysical characterization of protein–protein interactions (PPIs) that regulate biological functions in all organisms requires the use of recombinant proteins, many of which are difficult to produce in quantities required for these studies. Although in many cases one of the binding partners can be replaced by linear or cyclic peptides derived from the interfaces of PPIs (Benyamini and Friedler, 2010; Katz *et al.*, 2011), using peptides is limited by the cost, time and feasibility of synthesis. This can significantly restrict the ability to probe the contribution of both the length and composition of the peptide to the binding interaction, and is particularly pertinent for peptide sequences that are difficult or impossible to produce by solid phase synthesis. Furthermore, linear peptides are prone to proteolysis and challenging to introduce into cells, complicating their use in *in vivo* biological assays. Finally, in general, flexible molecules may have a lower affinity to their targets than their conformationally restrained variants (Ghosh *et al.*, 2005; Laudet *et al.*, 2007; DeLorbe *et al.*, 2009).

To overcome the limitations imposed by the properties of isolated peptides, a variety of different peptide display technologies have been developed (Table S1). Most of these rely on a small, highly soluble and thermostable protein core or ‘scaffold’ that can tolerate insertions into loop sequences or N-terminal extensions (Binz *et al.*, 2005; Reverdatto *et al.*, 2015; Škrlec *et al.*, 2015). Examples of such scaffolds include thioredoxin, Adhiron and Affimer (based on cystatins), kunitz domains, γ-S crystallin, Adnectin and Pronectin (fibronectin domains), Anticalin (from lipocalins), Fynomer (tyrosine kinase) and Knottins (Binz *et al.*, 2005; Hoffmann *et al.*, 2010; Lipovšek, 2011; Moody *et al.*, 2012; Schlatter *et al.*, 2012; Cappuccilli *et al.*, 2009; Tiede *et al.*, 2014; Škrlec *et al.*, 2015).

Several surface display scaffolds were designed to model broad surface-based PPIs and can tolerate mutations across an entire face of the protein (Binz *et al.*, 2005; Škrlec *et al.*, 2015). Some peptide display systems could potentially be used for development of medical applications including diagnostic assays, imaging and protein-based therapies (Binz *et al.*, 2005; Mintz and Crea, 2013; Haberkorn *et al.*, 2014; Reverdatto *et al.*, 2015; Škrlec *et al.*, 2015).

However, the majority of these display systems were designed to be used for *in vitro* directed evolution applications in iterative selection cycles to create novel binders with high affinity for the target protein and are often not well suited for biophysical interrogation of PPIs by different, complementary methods. Thus, these scaffolds were not suitable for our primary need in projects aimed at developing inhibitors against PPI involving linear epitopes.

**Fig. 1:**
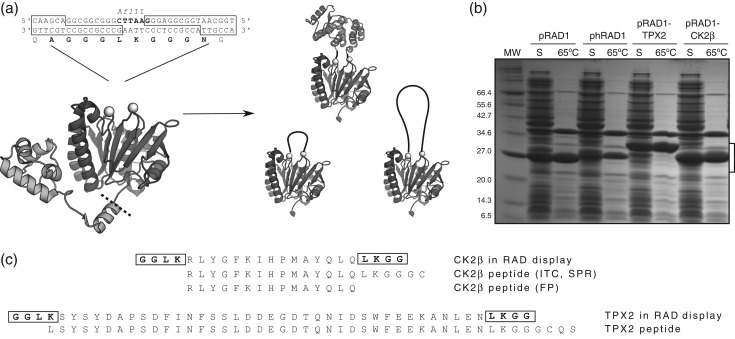
Overview of the RAD display system. (**a**) Sequence of the RAD expression plasmid LIC cloning site and schematic representation of peptides and a protein domain grafted on the RAD display system. The structure on the left shows full-length RadA protein. The N-terminal domain that is removed at the position indicated by a dashed line is shown in light gray and the C-terminal ATPase domain is shown in darker gray. The two spheres indicate the start and the end of the L2 loop where the displayed epitopes are inserted. The small structural diagrams illustrate how the system can tolerate insertions of different sizes, provided their N- and C-termini are in close proximity. (**b**) SDS-PAGE analysis of soluble fractions of the *E. coli* cell lysates after induction with IPTG before and after a 10 min 65°C heating step to precipitate most of bacterial proteins; the major remaining contaminant has not been identified, as it is easily removed in subsequent purification steps. The position of the RAD display protein is indicated by a square bracket. (**c**) Sequences of the CK2β and TPX2 peptides used in the binding assays, with RAD scaffold-derived sequences highlighted in bold.

To address this, we have developed a novel peptide display system in which the epitope of choice is grafted onto a scaffold based on the monomeric form of the RadA recombinase from *Pyrococcus furiosus*. The advantage of using this particular scaffold is the fact that RadA is a highly soluble medium-sized monomeric protein, which possesses high thermal stability despite not containing any cysteine residues. It has well-characterized structural organization (Fig. 1a) and is tolerant of surface mutations and deletions (Scott *et al.*, 2013). This RadA scaffold allows also for rapid production in *Escherichia coli* with high yields. In addition, a range of engineered expression constructs with differently tagged versions of this RadA-based scaffold (RAD) were created in order to facilitate immobilization of displayed peptides on solid surfaces or labeling with fluorescent dyes (Fig. S1).

To evaluate the functionality of the RAD display system, we have chosen to test two well-established protein interactions: TPX2/Aurora A kinase and CK2β/CK2α kinase-subunit complex (Fig. S2). Both complexes are therapeutically relevant and engage serine/threonine kinases that play important roles in carcinogenesis. As such, they serve as interesting model systems to demonstrate the usefulness of the RAD display as a tool for analyzing PPIs.

Aurora A kinase plays an important role in the mitotic spindle assembly and is overexpressed in tumor cells (Bischoff *et al.*, 1998). It is known to be dramatically activated by TPX2 protein, which binds to the Aurora A catalytic domain and translocates the kinase to the mitotic spindle during mitosis (Bischoff *et al.*, 1998; Kufer *et al.*, 2002; Eyers *et al.*, 2003). TPX2 has been shown to bind to Aurora A through its N-terminal segment with 2.3 nM affinity (Anderson *et al.*, 2007) and inhibitors of the TPX2/Aurora A interaction have been suggested to serve as potential therapeutic agents for cancer treatments (Asteriti *et al.*, 2010; Janecek *et al.*, 2016). A crystal structure of the TPX2/Aurora A peptide complex is available and shows that a conserved 43 residue long segment from the very N-terminus of TPX2 binds to the Aurora A catalytic domain through two separate antiparallel peptide stretches connected by a non-conserved flexible linker (Bayliss *et al.*, 2003) (Fig. S2a).

CK2α is a constitutively active kinase involved in cell growth, proliferation and suppression of apoptosis, and is often overexpressed in cancer cells (Landesman-Bollag *et al.*, 2001; Zhang *et al.*, 2015). The range of target substrates for CK2α is modulated by a scaffold protein, CK2β, that forms a reversible protein complex with an equilibrium binding constant (*K*_D_) of 5.4 nM (Martel *et al.*, 2002). A cyclic *Pc* peptide GCRLYGFKIHGCG derived from the C-terminal segment of the CK2β subunit has been shown to bind to CK2α with nanomolar affinity (*K*_D_ = 559 nM) and to act as an allosteric activator of CK2α (Raaf *et al.*, 2013) (Fig. S2b). The crystal structure of the complex between CK2α kinase and the *Pc* peptide revealed that the peptide interacted with the N-lobe of the kinase in a manner analogous to the interaction with the full-length CK2β subunit (Lolli *et al.*, 2012). Notably, the linear form of the *Pc* peptide was found to be a less potent competitive inhibitor than the constrained *P*c peptide and inhibited the formation of CK2β/CK2α complex with the IC_50_ of 30 μM, which was 10 times less potent than the cyclic *P*c peptide constrained by a disulfide bond (IC_50_ 3 μM) (Laudet *et al.*, 2007).

Using three orthogonal biophysical techniques, we demonstrate that both long and short peptides grafted on the RAD scaffold are easily produced in bacteria and bind to their natural targets with affinities comparable to or better than the equivalent linear peptides.

To further prove the usefulness of the scaffold system in analyses of PPIs, we have used the RAD system for alanine scanning of the TPX2/Aurora A interaction and have identified hotspots on the TPX2 that contribute to the high-affinity Aurora A kinase binding activity, demonstrating that this display system can be used as a low cost surrogate for synthetic peptides and full-length proteins in both biophysical and biochemical studies of PPIs.

## Results

### RAD scaffold design

To develop a novel peptide display system, we have taken advantage of our observation that the thermostable recombinase RadA from *P. furiosus* can tolerate sequence diversity on its so-called DNA binding L2 loop (Fig. 1a) (Marsh *et al.*, 2016). The monomeric version of RadA recombinase missing 107 N-terminal residues retains the solubility and thermostable properties of the full-length protein (Scott *et al.*, 2013). Due to its high isoelectric point, the untagged globular ATPase domain of RadA is easily purified from *E. coli* using cation exchange chromatography followed by size exclusion chromatography (SEC), while affinity-tagged versions are well suited for rapid processing of multiple constructs in parallel, for example for screening purposes. Additionally, the high thermal stability of RadA allows for fast initial enrichment of the protein by a simple heat treatment, where the bacterial lysate is heated at neutral pH to 65°C for 10 min and the precipitated *E. coli* proteins are removed by centrifugation, resulting in a high level of purity prior to chromatography (Fig. 1b). This feature can be particularly useful for rapid screening of RAD display variants for binding activity.

Our previous work on RadA has shown that its so-called L2 loop of can be deleted without affecting the protein stability (Scott *et al.*, 2013). This loop is involved in binding to single- and double-stranded DNA (Chen *et al.*, 2008) and disordered in most structures of RadA and its orthologs. Guided by analysis of various RadA crystal structures (Pellegrini *et al.*, 2002; Shin *et al.*, 2003; Wu *et al.*, 2005), we chose residues A287 and G302 as the anchor points for peptide insertion. As shown in the crystal structure of RadA (Fig. 1a), these residues are close to each other in three-dimensional space but also somewhat removed from the globular core such that additional residues are unlikely to interfere with folding and stability of RadA. In order to further minimize the effect of the scaffold on the peptide properties, we generated short flexible linkers on both sides of the peptide insertion site and designed the DNA expression construct to be compatible with ligation-independent cloning (LIC) (Aslanidis and de Jong, 1990). This allows for insertion of any epitope sequence using synthetic DNA oligonucleotides that are annealed to form dsDNA with compatible 5′ssDNA overhangs into the LIC-ready vector (Figs. S1 and S3).

Expression of all constructs in bacteria is regulated by the T7*lac* promoter and designed to facilitate flexibility in purification and biophysical applications by including options for addition of an N-terminal His_6_-tag and a unique cysteine that allows for site-specific labeling and directional immobilization through the very N-terminus of the display protein. Plasmids were constructed to express untagged, His_6_-tagged, His_6_-Cys-tagged, Cys-tagged and Cys-Strep-tagged versions (Fig. S1). Further, a final expression plasmid was created for production of proteins with a high-affinity N-terminal double-His_6_-tag, with the two His-tags separated by an AviTag for site-specific biotinylation by BirA biotin transferase (Schatz, 1993).

### Production of RAD display proteins in *E. coli*

For the validation of the RAD peptide display system, we created various RAD display constructs for the production of scaffolds carrying Aurora A and CK2a binding epitopes from TPX2 and CK2β, respectively. DNA sequences encoding for residues 7–43 of human TPX2 (TPX2) (Fig. 1c) and for residues 186–200 of human CK2β (CK2β) were produced as synthetic oligonucleotides (Fig. 1c). Double-stranded DNA fragments were then assembled by thermal denaturation and annealing from contiguous overlapping complementary primers (Table S2) and then inserted into different pRAD vectors (Figs. S1 and S3) using LIC (Aslanidis and de Jong, 1990; Aslanidis *et al.*, 1994). In the next step, the chimeric proteins were tested for soluble expression in *E. coli* to thereby determine the tolerance of the RAD scaffold for both long and short peptide sequence insertions. Scaffolds displaying TPX2 and CK2β peptides and their variants could be produced in *E. coli* and rapidly purified using a heat treatment step (Fig. 1b) followed by cation exchange and SEC. On average ~10 mg of pure protein could be produced from 1 l bacterial culture. All RAD display proteins used in this work are listed in the Supplementary Material (Table S3).

For the interaction analyses, full-length CK2α kinase (CK2α) and the kinase domains of Aurora A (Aurora A_WT_ and Aurora A_D274N_) were produced in *E. coli* and purified to homogeneity.

### Analysis of complex formation using sedimentation velocity analytical ultracentrifugation and analytical SEC

To demonstrate the possibility of studying complex formation between the kinases and peptides displayed on the RAD scaffold, protein mixtures were subjected to sedimentation velocity analytical ultracentrifugation (SV AUC) with UV absorbance detection at 280 nm.

**Fig. 2:**
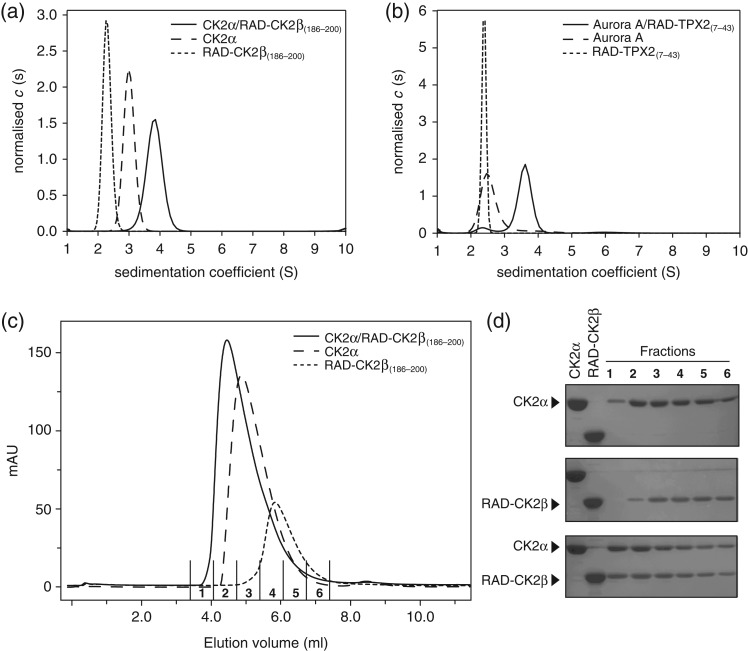
Sedimentation velocity AUC and size exclusion chromographic analyses interactions between RAD display epitopes and the interacting kinases. (**a**) AUC analysis of CK2α kinase (line with long dashes), RAD-CK2β_(186–200)_ (short dashes) and their complex (solid line). (**b**) AUC analysis of Aurora A_D274N_ kinase (long dashes), RAD-TPX2_(7-43)_ (short dashes) their complex (solid). (**c**) Analytical size exclusion chromatographic (SEC) analysis CK2α kinase (long dashes), RAD-CK2β_(186–200)_ (short dashes) and their complex (solid line). Fractions collected for SDS-PAGE analysis are numbered in the chromatogram (1–6). (**d**) SDS-PAGE analysis of fractions 1–6 from the three SEC analyses in (c). In each gel, purified CK2α kinase and RAD-CK2β_(186–200)_ are loaded in the left-most lanes as markers.

SV AUC is a widely used technique to study proteins and protein interactions and provides information on the size and shape of molecules in solution (Schuck *et al.*, 2001; Schuck, 2013). Figure 2 shows results from the hydrodynamic measurements for the kinases and RAD display proteins and their corresponding complexes. Fitting the sedimentation velocity profiles with a single species Lamm equation model revealed that both CK2α kinase and RAD-CK2β sediment as single species with *s*-values of 3.0 S and 2.3 S, respectively (Fig. 2a, Fig. S4a and b). The analysis of the boundary spreading of the data shown in Fig. 2a shows molar masses of 41.5 kDa for CK2α kinase and 28.0 kDa for RAD-CK2β, which is virtually identical to the molar mass of the monomeric CK2α of 39.9 kDa and RAD-CK2β of 28.1 kDa calculated from the amino acid composition of the expression constructs. In contrast, for the CK2α/RAD-CK2β mixture, we observed a single species that sedimented with an *s*-value of 3.8 S (Fig. 2a, Fig. S4c). Sedimentation data analysis resulted in an estimated molar mass of 59.3 kDa for the 3.8 S species, which strongly indicated that the proteins formed a 1:1 complex in solution (calculated molar mass of the CK2α/RAD-CK2β complex is 68 kDa).

Similarly, SV AUC experiments confirmed formation of a hetero-dimeric protein complex between Aurora A_D274N_ and RAD-TPX2 in solution defined by the appearance of a larger species with a sedimentation coefficient of 3.6 S (Fig. 2b, Fig. S4d–f) compared with the species profiles obtained in the experiments with individual proteins alone. The analysis of the boundary spreading in the sedimentation velocity experiments with the Aurora A_D274N_ and RAD-TPX2 mixture resulted in a molar mass estimate of 54.7 kDa, suggesting that the observed 3.6 S species is a hetero-dimeric complex. A small (<10%) population of species was also observed to sediment at 2.5 S (Fig. 2b, Fig. S4f), which could be attributed to the unbound monomeric kinase or display proteins.

In contrast, Aurora A_D274N_ kinase domain and RAD-TPX2 sediment with *s*-values of 2.6 S and 2.5 S, respectively (Fig. 2b, Fig. S4d and e). This resulted in an estimated molar mass of 34.5 kDa for Aurora A_D274N_ kinase and 31.9 kDa for RAD-TPX2 display, which was in line with theoretical molar masses of 33.8 and 30.4 kDa for the kinase domain and RAD display, respectively.

In order to demonstrate that the stoichiometry of interactions between the RAD display protein and their cognate binding partners can be analyzed using more commonly available techniques, we also subjected CK2α kinase, RAD-CK2β and an equimolar mixture to analytical size exclusion over a Superdex 75 5/150 column (Fig. 2c and d), Both CK2α kinase and RAD-CK2β elute as single species at 1.48 and 1.72 ml, respectively. Calibration controls (Fig. S5) determine that these elution volumes are equivalent to molecular weights of 45.7 and 22 kDa for CK2α kinase and RAD-CK2β, respectively, consistent with the calculated and AUC determined molecular weights (above). The mixture of the two proteins eluted as a single species with a peak at 1.32 ml, equivalent to a molecular weight of 74.3 kDa indicating that CK2α kinase and RAD-CK2β form a 1:1 complex consistent with the AUC data above (Fig. 2, Fig. S4).

### Comparison of linear or displayed peptides for binding to their targets

We next compared the affinities of TPX2 and CK2β to their corresponding kinase partners both as displayed on the RAD scaffold and as isolated peptides. Sequences of the peptides were identical between the display versions and the linear peptides, but suitable chemical handles were introduced to the ends of the peptides to enable site-specific labeling or immobilization. Whereas short linear CK2β peptide variants were synthesized chemically (Fig. 1c, Fig. S6a), the significantly longer TPX2 peptide has proven difficult to synthesize using standard solid phase chemistry and was produced in *E. coli* using the KSI fusion system (Kuliopulos and Walsh, 1994) (Fig. 1c, Fig. S6b).

To demonstrate that the RAD system is suitable for application to the commonly used affinity determination techniques, isolated peptides and RAD proteins carrying the same peptide sequences were used to determine and compare their affinities to CK2α and Aurora A_D274N_ kinase using three complementary methods for determination of the equilibrium binding constant (*K*_D_): isothermal titration calorimetry (ITC), surface plasmon resonance (SPR) and fluorescence anisotropy (FA). The three analytical methods are standard techniques and have their different strengths, weaknesses and analyte requirements and provide complementary data on the interactions.

ITC measures the heat that is released or absorbed during a binding event, and allows for the determination of the thermodynamic parameters for the interaction. ITC requires significant quantities of both interacting components, with the component to be titrated into the sample cell needing to be stable and soluble at reasonably high concentrations.

By contrast, SPR analysis demands less of the sample, but requires one of the binding partners to be immobilized on the dextran matrix on the sensor surface, ideally in a defined orientation. Additionally, the immobilized component needs to be sufficiently stable to withstand repeated regeneration cycles without losing its activity.

FA assays can be performed in solution at very low concentrations of analytes and in very small volumes and with high-throughput, compared with the ITC and SPR that are low- and medium-throughput methods, respectively. The disadvantage of the FA method is that it requires one binding partner to be fluorescently labeled, preferably in a homogenous fashion.

### ITC analyses

To evaluate the use of RAD system for affinity determination, standard ITC experiments were conducted at 25°C. To determine the basic thermodynamic binding parameters, purified CK2β and TPX2 peptides and their RAD display variants were titrated to CK2α or Aurora A_D274N_ kinase domain (Fig. S7), respectively.

**Fig. 3:**
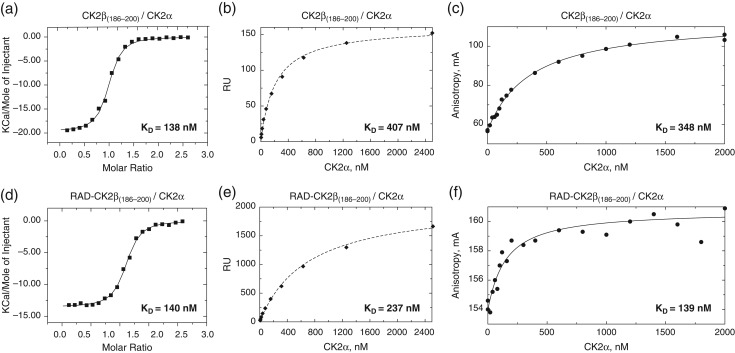
Characterization of the CK2α kinase binding to synthetic CK2β_(186–200)_ peptide or RAD-CK2β_(186–200)_ display by ITC, SPR and FA. (**a**) CK2α kinase binding to the synthetic linear CK2β_(186–200)_ peptide measured by ITC. (**b**) SPR analysis of CK2α kinase binding to the synthetic linear CK2β_(186–200)_ peptide immobilized on the surface of a CM5 chip using data at 200 s post-injection. (**c**) Analysis of CK2α kinase binding to the synthetic linear Fluorescein-CK2β_(186–200)_ peptide by FA. (**d**) CK2α kinase binding to cRAD-CK2β_(186–200)_ measured by ITC. (**e**) SPR analysis of the CK2α kinase binding to cRAD-CK2β_(186–200)_ immobilized on the surface of CM5 chip using data at 200 s post-injection. (**f**) Analysis of the CK2α kinase binding to the Alexa Fluor 488-labeled cRAD-CK2β_(186–200)_ by FA.

**Fig. 4:**
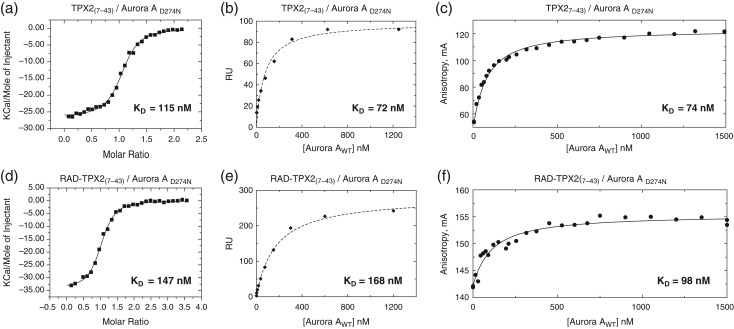
Characterization of the Aurora A_D274N_ kinase binding to TPX2_(7–43)_ peptide or RAD-TPX2_(7–43)_ by ITC, SPR and FA. (**a**) Aurora A_D274N_ kinase binding to the linear TPX2_(7–43)_ peptide measured by ITC. (**b**) SPR analysis of Aurora A_D274N_ kinase binding to the linear TPX2_(7–43)_ peptide immobilized on the surface of a CM5 chip. (**c**) Analysis of Aurora A_D274N_ kinase binding to Alexa Fluor 488-TPX2_(7–43)_ peptide by FA. (**d**) Aurora A_D274N_ kinase binding to the cRAD-TPX2_(7–43)_ display protein measured by ITC. (**e**) SPR analysis of Aurora A_D274N_ kinase binding to the cRAD-TPX2_(7–43)_ display protein immobilized on a CM5 chip. (**f**) Analysis of Aurora A_D274N_ kinase binding to Alexa Fluor 488-labeled cRAD-TPX2_(7–43)_ display protein by FA.

In the ITC assay, both display peptides performed with the same efficacy in the binding assays as their corresponding linear versions (Table I). For the CK2β/CK2α interaction, the binding affinity was 138 and 140 nM for the peptide and RAD-CK2β display, respectively (Fig. 3a and d, Fig. S7 and Table I). The binding constant *K*_D_ for the Aurora A_D274N_ interaction with RAD-TPX2 display or TPX2 peptide was found to be 147 and 115 nM, respectively (Fig. 4a and d, Fig. S7 and Table I). Thus, these ITC experiments demonstrated the ability of the scaffold to present peptide epitopes of different lengths without loss of the binding affinity to the target protein compared with the linear peptides.

**Table I.** Summary of the affinity data

Complex	Method	Peptide (nM)	RAD peptide (nM)
TPX2_(7–43)_/Aurora A_D274N_	ITC	115 ± 10	147 ± 10
	SPR	72 ± 14	168 ± 14
	FA	74 ± 5	98 ± 19
CK2β_(186–200)_/CK2α	ITC	138 ± 13	140 ± 19
	SPR	407 ± 50	237 ± 17
	FA	348 ± 27	139 ± 48

Equilibrium dissociation constants (*K*_D_) for CK2β_(186–200)_/CK2α kinase and TPX2_(7–43)_/Aurora A_D274N_ kinase determined for linear peptides and peptides presented by the RAD display using different methods. The data were typically derived from a single titration experiment and the confidence interval (±*K*_D_) was estimated from the fitting.

ITC measurements suggested that the RAD scaffold did not promote significant peptide pre-organization as the binding energetics show no entropic advantage for either of the test complexes. For both complexes, we observed very similar affinities between the linear parent peptide and the peptide grafted on the RAD scaffold (Table I). For both kinases, unfavorable entropic contributions were observed for the interaction with the displayed peptides consistent with the observations made by others (DeLorbe *et al.*, 2009; Udugamasooriya and Spaller, 2008). While in the case of Aurora A/TPX2 interaction we observed only minor differences in relative enthalpies and entropies (Fig. S8a–d), for CK2α, the interaction with the RAD display showed a larger entropic penalty than when binding to the peptide.

### SPR analyses

SPR requires one of the two interacting partners to be immobilized to the sensor surface without significant loss while the other partner (analyte) is flowed over the surface. The interaction is detected by the change in the refractive index of the surface as the analyte binds to the immobilized partner. For the interaction to occur, the immobilized partner needs to be positioned in a suitable orientation and away from the dextran matrix on the surface so as not to be sterically hindered. With linear peptides this can be difficult to achieve without introducing additional linkers to the end of the peptide that is being immobilized. A larger scaffold displaying the peptide would ensure the binding epitope is accessible on the surface.

With the RAD display system, we have overcome these possible limitations by introducing a unique cysteine residue in the very N-terminus of the protein to enable site-specific immobilization using maleimide chemistry. As the N-terminus of the domain is on the opposite side from the inserted peptide epitope, the displayed peptide will be positioned well away from the SPR surface and exposed to the analyte. Also, the immobilized partner needs to be stable enough to allow for the bound analyte to be dissociated, typically under relatively harsh conditions and the surface regenerated in order to bind to the analyte again.

We tested the promise of RAD to facilitate SPR measurements using both of our test systems. The CK2β peptide presented by the cRAD system had a 3-fold higher affinity (*K*_D_ = 237 nM) for the CK2α kinase compared with the linear peptide immobilized on the CM5 chip surface (*K*_D_ = 407 nM) (Table I, Fig. 3b and e, Fig. S9a and b). The synthetic peptide had the same sequence as the RAD-displayed epitope, with two additional residues in its C-terminus (Gly-Cys) to enable covalent coupling to the surface. It seems that grafting of the short CK2β peptide onto the RAD scaffold brought advantage for the SPR assay and resulted in a more comparable *K*_D_ value to what was determined by ITC.

In the SPR assays, Aurora A/TPX2 interactions demonstrated slow dissociation, but analysis of the kinetic data was not possible using the expected 1:1 binding model and could only be modeled using a heterogenous ligand model. It is unclear where this heterogeneity originates from, both the free and RAD-displayed TPX2 demonstrate similar behavior (Fig. S9e and f and associated figure legend), it seems to reflect the properties of the peptide, possibly different solution conformations or alternative binding modes. Analyzing the data using a steady-state model, TPX2 peptide displayed on the RAD scaffold bound Aurora A_D274N_ with an affinity comparable to the linear TPX2 peptide. The 45 amino acid long linear TPX2 peptide bound to Aurora A_D274N_ with *K*_D_ values within an ~2-fold range of the values observed for RAD-displayed peptide (Table I, Fig. 4b and e, Fig.  S9c and d). These values were also comparable to the ones obtained by ITC (Table I).

### FA measurements

The FA method allows rapid and quantitative analysis of molecular interactions in solution. This technique requires one of the binding partners to be labeled with a fluorescent tracer. The label is preferably attached to the smaller binding partner in order to maximize the dynamic range of the assay. Both RAD-TPX2 and RAD-CK2β display proteins were labeled with Alexa Fluor 488-maleimide using the unique N-terminal cysteine introduced by the cRAD scaffold. For the CK2β/CK2α interaction, the binding affinity was determined to be 348 nM for the peptide and 139 nM for the RAD-CK2β display (Table I, Fig. 3c and f). The ~3 times weaker affinity of the fluorescein labeled linear CK2β peptide compared with the display may reflect the negative effect of the fluorescent tracer being located proximally to the short peptide during the formation of the complex and is in line with the similar effect seen in SPR. This could possible be overcome by using a longer peptide, but we deliberately used the core peptide epitope for the FA experiment as this would be expected to yield largest difference in FA upon binding. It is worth noting that affinities measured for RAD-displayed CK2β peptide are more consistent (139–237 nM) between the different experimental techniques than those measured for the peptide (138–407 nM).

The binding affinity for the TPX2/Aurora A_D274N_ complex was found to be similar for the linear TPX2 peptide and RAD-TPX2 (*K*_D_ = 74 nM for the linear peptide and *K*_D_ = 98 nM for the RAD display). Even with the comparatively narrow dynamic range of the FA assay while using fluorescently labeled RAD display as tracers, the obtained *K*_D_ values were comparable to those using labeled linear peptide, which had a much broader dynamic range (Table I, Fig. 4c and f).

### Hotspots in TPX2: Aurora A binding site

To resolve the residues or ‘hot spots’ in the TPX2 peptide that drive affinity for Aurora A, a double His-tagged RAD scaffold was utilized to produce seven different alanine mutants of TPX2 in parallel, which were then used for affinity determination by a direct binding assay using Bio-Layer Interferometry (BLI) and by the FA competition assay.

**Fig. 5:**
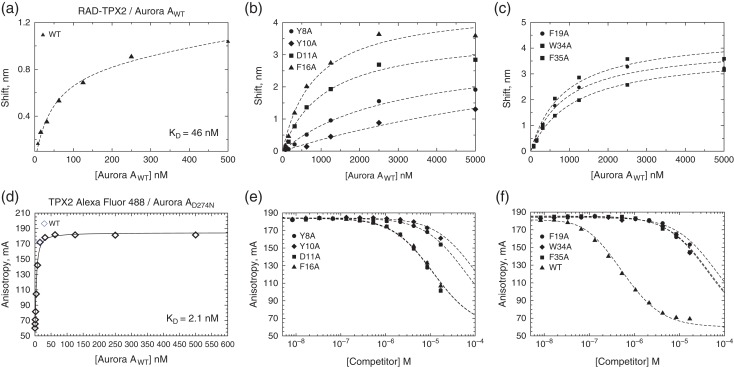
Identification of hot spots for TPX2:Aurora A interaction. (**a**) BLI analysis of Aurora A_WT_ binding to wild-type RAD-TPX2_(7–43)_. (**b**) BLI analysis for the Aurora A_wt_ binding the RAD-TPX2_(7–43)_ alanine mutants Y8A, Y10A, F16A and D11A. (**c**) BLI analysis for the Aurora A_wt_ binding to the RAD-TPX2_(7–43)_ alanine mutants F19A, W34A and F35A. (**d**) Determination of the binding affinity between Alexa Fluor 488-TPX2 peptide and Aurora A_WT_ by FA. (**e**) FA competition assay for the binding of Aurora A_wt_ to the wild-type RAD-TPX2_(7–43)_ display scaffold and alanine mutants Y8A, Y10A, F16A and D11A. (**f**) FA competition assay for the binding of Aurora A_wt_ to the wild-type RAD-TPX2_(7–43_) display scaffold and alanine mutants F19A, W34A and F35A.

RAD-TPX2 mutants, purified by IMAC chromatography and buffer exchanged into the assay buffer, were used in direct binding tests using BLI (Fig. 5a–c; Figs. S10a and S11). Since the His-tagged RAD-TPX2 was used for immobilization on the BLI surface, in this assay, the His_6_-tagged Aurora A_D274N_ construct was replaced by the untagged wild-type Aurora A kinase domain construct (Aurora A_WT_, Fig. 5a).

These BLI experiments were able to determine affinities of all the TPX2 mutants (Fig. 5b; Table II). Mutations of Y8 and Y10 to alanines had the most negative impact on the interaction, but removal of any of these interacting side chains resulted in at least 20-fold reduction in the affinity between TPX2 and Aurora A, demonstrating their critical roles in this complex.

**Table II.** Affinities of TPX2 mutants towards wild-type Aurora A

TPX2 mutation	Assay	
	FA	BLI
	*K*_D_ (μM)	*K*_D_ (μM)
WT	0.002	0.046
Y8A	>3.2	2.8
Y10A	>3.2	11.0
D11A	>3.2	1.1
F16A	0.3	0.82
F19A	1.77	0.87
W34A	1.17	0.84
F35A	1.11	1.1

Dissociation constants (*K*_D_) for different RAD-TPX2_(7–43)_ mutants, determined by using FA competition assay and by measuring direct binding with BLI.

To confirm the results of the BLI analyses, we used a competition FA assay to determine affinities for each of the seven alanine mutants of the RAD-TPX2 in solution and compare these with the values obtained with the wild-type RAD-TPX2 (Fig. 5d–f).

In order to perform competition binding experiments, we assessed the affinity of the Alexa Fluor 488-labeled TPX2 peptide for wild-type Aurora A kinase domain. FA measurements were performed by titrating increasing concentrations of the kinase into constant concentration of labeled TPX2 peptide (10 nM). This yielded a *K*_d_ of 2.1 nM (Fig. 5d), in good agreement with previously reported data (Anderson *et al.*, 2007). This was confirmed by ITC (Fig. S12) and clearly shows that the affinity of the TPX2 to Aurora A is altered by the D274N mutation. D274 is part of the so-called DFG motif in Aurora A and coordinates the Mg^2+^ ion in the ATP binding site. It is likely that abolishment of Mg^2+^ binding has an allosteric effect on TPX2 binding site, resulting in the significant difference we have observed in the binding affinities. In the BLI experiments Aurora A has a ~20 fold weaker affinity to RAD-TPX2 compared to the values determined in the FA assay using fluorescently labeled linear TPX2 peptide, suggesting that the BLI sensor surface may have a negative effect on this interaction.

In the FA competition assay, the labeled wild-type TPX2 peptide was competed by increasing concentrations of each of the eight mutant RAD-TPX2 proteins. These experiments revealed that TPX2 mutants Y8A and Y10A, along with D11A, had the lowest affinities for the Aurora A_WT_ kinase domain (Fig. 5e; Table II). Mutation of these conserved tyrosines to alanines decreased the affinity of TPX2 by a factor >300 (Table II). This was consistent with the results obtained in the BLI experiments and was in line with the observations by Eyers and Maller that the hydrophobic residues Y8 and Y10 were critical for activation of Aurora A kinase by TPX2 (Eyers and Maller, 2004). Effects of the other mutated residues in TPX2 affinity were in similarly good agreement with the BLI data.

## Discussion

To extend the tool repertoire for the PPI studies, we have designed a new peptide display system, termed ‘the RAD display’, which is based on the RadA recombinase from *P. furiosus.* The thermal stability and design of the novel scaffold allows for high-level production and rapid purification of soluble display proteins in *E. coli*. The RAD scaffold was demonstrated to be a well-expressed and well-behaved protein that tolerates both short and long peptide insertions as long as 45 amino acids in RAD-TPX2. Indeed, the TPX2 peptide insertion is the longest reported one, along with the 42 residues displayed by the Affimer scaffold (Hoffmann *et al.*, 2010). We have also been able to display a complete protein in this system, with soluble expression and purification of T4 lysozyme containing RAD scaffold (data not shown). In this case, the thermal stability of the whole construct was not retained, as would be expected whenever a fully folded domain is inserted into the scaffold. We have also expressed number of epitopes from structured part of TGF-β growth factors successfully in this system, demonstrating the ability of the RAD scaffold to facilitate soluble expression and stability of relatively hydrophobic epitopes as well (Fig. S13). Using affinity-tagged proteins, parallel purification can be easily achieved and also allows for easy and rapid ‘semi-pure’ screening systems to be developed without the need for multi-step protein purification (Fig. 1, Fig. S10a).

As the displayed peptide is inserted in the middle of the scaffold it is likely to be more protected from proteolysis when compared with fusion of such peptide to either terminus of the protein, as a flexible ‘tail’. Further, stability of the scaffold will also be beneficial for long-term storage of the protein as well as for the development of assays where the scaffold is likely to be exposed to harsh conditions, such as regeneration of protein on an SPR sensor chip.

A unique cysteine in the scaffold allows for labeling in a controlled and uniform manner using the well-established maleimide chemistry. Labeling with biotin, either chemically through the cysteine or by enzymatic modification of the AviTag would allow interaction of the scaffold with streptavidin, and the utilization of a wide variety of technologies that take advantage of this high-affinity interaction for screening, binding, purification and selection. Labeling with fluorescent dyes will allow the protein to be used for various fluorescence-based binding assays, as demonstrated by our FA binding assays.

The scaffold allows presentation of the peptide in an oriented fashion due to a spacer on the RAD display that separates the epitope from the labeling or immobilization site in the N-terminus of RAD display. We have shown through several complementary techniques that epitopes in the RAD display scaffold interact with their targets in a similar manner to isolated peptides. When analyzing the relatively short CK2β peptide binding to CK2α kinase, the results with the RAD display were more consistent across the different experimental techniques, suggesting that for shorter epitopes immobilization or derivatization of the peptide influences its properties significantly, resulting in less accurate measurements.

The LIC site in the various scaffold expression plasmids facilitates rapid generation of constructs using overlapping oligonucleotides. As there appear to be few constraints with respect to the inserted sequence, it is easy to construct libraries of epitopes, with mutations in specific positions of the target sequence. Such libraries (or panels) of variant epitopes could be used for the development of higher affinity binding variants of the original sequence or to probe the specificity of residues in particular positions of the epitope, similar to what we have by alanine scanning of TPX2 epitope.

In conclusion, the RAD display serves as a flexible and robust platform for the selection or characterization of peptides in a variety of applications ranging from basic research tool to drug discovery. We envisage that RAD display could be useful in expression of binding epitopes in mammalian cells as well, for functional analysis of loop epitopes, by providing a stable platform to ensure otherwise completely unstructured peptides are not immediately destroyed. The scaffold could be tagged with localization signals to direct it to a desired subcellular location like the nucleus. Given that the RAD scaffold tolerates a wide range of inserts, it could also be used as a platform to display epitope libraries varying different length and composition for the identification of novel binders or for the determination of affinity determinants in a high-throughput fashion.

## Materials and methods

### Construction of RAD display vectors

All oligonucleotide sequences are listed in Table S2. Point mutation K144A in the active site mutation of the *P. furiosus* RadA (*Pf*RadA, Uniprot: O74036) ATPase domain was created using overlapping PCR with primer pairs RAD-K144A-F and RAD-K144A-R and subcloned into pBAT4 expresson plasmid using the NcoI and XhoI sites to create *Pf*RadA K144A. Residues 108–350 of this construct were amplified by PCR from pBAT-PfRadA3 K144A plasmid in two stages to introduce the LIC site in the place of the L2 loop using primers pRAD and pRAD-LIC2, and HAT2 and pRAD-LIC1 primers. For constructs containing a free cysteine for labeling, pRAD1 primer was replaced with primer pcRAD1. Amplified fragments were digested with NcoI and XhoI and ligated into similarly digested pBAT4 or pHAT vectors for untagged and His-tagged versions, respectively.

All constructs were confirmed correct by sequencing. Vector maps with sequences of the cloning site for display peptides are shown in the Supplementary Material.

### Cloning of RAD display constructs for TPX2 and CK2β peptides

Peptide sequences to be displayed on the RAD scaffold were reverse translated and codon optimized for *E. coli* expression. Redundancy in the codons was utilized to maximize the specificity and stability of dsDNA assembly from contiguous, overlapping, complementary synthetic oligonucleotides and 5′ and 3′ ssDNA overhangs complementary to the LIC-ready vector were added to the oligonucleotides constituting the 5′ and 3′ ends of the insertion coding sequence (Table S2). The LIC pRAD-TPX2 expression plasmid was digested with NcoI and NotI and ligated into the pHAT plasmid digested with the same enzymes to produce a His_6_-tagged construct of RAD-TPX2. The resulting plasmid was used as a template for PCR using primer pair pRAD-TPX2-F, pRAD-TPX2-R or phRAD-TPX2-F and pRAD-TPX2-R. Alanine mutant variants of this construct were then produced by site directed mutagenesis using the pRAD-TPX2/(X#A) series of primers. PCR products were digested with BspHI and XhoI for ligation with pHAT4-haXrn6 digested with NcoI and XhoI. Alanine mutant variants of this construct were then produced by site directed mutagenesis using the pRAD-TPX2/(X#A) series of primers. TPX2 mutants for alanine scanning were inserted into RAD scaffold with His_6_-Avi-His_6_-tag to facilitate stable immobilization with anti-penta-His sensors for BLI.

### Expression of RAD display proteins

RAD display scaffolds with or without target peptide sequences were expressed in BL21(DE3) cells containing pUBS520 plasmid (Brinkmann *et al.*, 1989) in 2xYT media supplemented with 100 μg/ml ampicillin and 25 μg/ml kanamycin. Cultures were grown at 37°C to OD_600_ of 0.8 and induced with 0.4 mM IPTG. Protein was expressed for 3 h at 37°C and cells were harvested by centrifugation at 4000×*g* for 10 min at 4°C. Cell pellets were stored at −80°C.

### Purification of untagged RAD display proteins

Frozen cell pellets were thawed, resuspended in 5 ml/g of cells in 50 mM Hepes pH 7.4, 150 mM NaCl and lysed by three passages through an Emulsiflex C5 homogenizer. Lysates were heated for 10 min at 65°C before centrifugation at 30 000×*g* at 4°C for 30 min to pellet precipitated *E. coli* proteins. The supernatant was dialyzed against 10 mM MES pH 6, 0.5 mM EDTA, purified by cation exchange chromatography over a 5-ml HiTrap SP column (GE Healthcare) and eluted in a gradient from 0 to 1 M NaCl in elution buffer (10 mM MES pH 6, 0.5 mM EDTA). Peak fractions were pooled, concentrated and further purified by SEC over Superdex 75 16/60 HiLoad (GE Healthcare) column equilibrated with size exclusion buffer 10 mM MES pH 6, 0.5 mM EDTA, 200 mM NaCl, 1 mM DTT. Peak fractions were pooled, concentrated to 5–7 mg/ml, flash frozen in liquid nitrogen and stored at −80°C.

### Expression and purification of His-tagged RAD display proteins

Frozen cell pellets were resuspended in 5 ml/g of cells in water and lysed by three passages through an Emulsiflex C5 homogenizer. Lysates were heated for 10 min at 65°C before centrifugation at 30 000×*g* at 4°C for 30 min to pellet precipitated *E. coli* proteins. The supernatant was adjusted to 50 mM Tris pH 8, 500 mM NaCl, 1 mM DTT, 40 mM imidazole, applied on 0.5 ml Ni Sepharose beads (GE Healthcare), washed with 30 ml 50 mM Tris pH 8, 500 mM NaCl, 1 mM DTT, 40 mM imidazole and eluted in 5 ml buffer containing 50 mM Tris pH 8, 500 mM NaCl, 1 mM DTT, 600 mM imidazole.

For BLI experiments and competition FA assays eluted His-tagged proteins were buffer exchanged into the BLI buffer and stored at 4°C. For other binding assays proteins were further purified by SEC over HiLoad Superdex 75 16/60 column (GE Healthcare) equilibrated with size exclusion buffer 10 mM MES pH 6, 0.5 mM EDTA, 200 mM NaCl, 1 mM DTT. Peak fractions were concentrated to 5–7 mg/ml, flash frozen in liquid nitrogen and stored at −80°C.

### Expression and purification of CK2α kinase

Human CK2α kinase (residues 2-329) was expressed in BL21(DE3) cells containing pUBS520 plasmid in 2xYT media supplemented with 100 μg/ml ampicillin and 25 μg/ml kanamycin. About 4 l of cell culture were grown at 37°C until an OD_600_ of 0.6 when the temperature was lowered to 25°C for 1 h before induction with 0.4 mM IPTG. Protein was expressed for 18 h overnight at 25°C and the cells were harvested by centrifugation at 4000×*g* for 10 min at 4°C. Cell pellets were stored at −80°C until required when they were resuspended in 5 ml/g of cells in 100 mM MES pH 6, 0.5 mM EDTA and lysed by three passages through an Emulsiflex C5 homogenizer. Lysate was cleared by centrifugation at 40 000×*g* at 4°C for 30 min. Clarified lysate was purified over a 5 ml HiTrap SP column (GE Healthcare) and eluted by a gradient to from 0 to 1 M NaCl in 100 mM MES pH 6, 0.5 mM EDTA over 30 column volumes. To stabilize the protein, NaCl was added to 1 M to the pooled elution peak fractions, which were consequently dialyzed overnight against 100 mM MES pH 6, 0.5 M NaCl, 0.5 mM EDTA. Protein was further purified by SEC over a Superdex 75 16/60 HiLoad column (GE Healthcare) equilibrated with 20 mM Tris-HCl pH 8, 500 mM NaCl, 2 mM DTT. CK2α kinase required a buffer containing at least 500 mM NaCl to remain soluble. Purified CK2α had a strong absorption peak at 260 nM even after the size exclusion step, which we attributed to the possible contamination by nucleotides bound to the active site. The peak disappeared though after prolonged protein dialysis in the size exclusion buffer overnight at 4°C. Peak fractions were pooled, concentrated to 10 mg/ml and flash frozen in liquid nitrogen for storage at −80°C.

### Expression and purification of Aurora A

Kinase dead human Aurora A_D274N_ mutant (residues 122-403) was cloned into pHAT5 vector in frame with a C-terminal 6xHis-tag. Protein was expressed in BL21(DE3) cells containing pUBS550 plasmid cultured in 2xYT media supplemented with 100 μg/ml ampicillin and 25 μg/ml kanamycin. About 4 l of cell culture were grown at 37°C until an OD_600_ of 0.6 when the temperature was lowered to 25°C for 1 h before induction with 0.4 mM IPTG. Protein was expressed for 18 h overnight and the cells were harvested by centrifugation. Cell pellets were stored at −80°C until required when they were resuspended in 5 ml/g of cells in 50 mM Tris-HCl 8, 0.5 M NaCl, 20 mM imidazole and lysed by 3 passages through an Emulsiflex C5 homogenizer. Lysate was cleared by centrifugation at 30 000×*g* at 4°C for 30 min, applied on a 5 ml HisTrap HP column (GE Healthcare). Protein was eluted over 30 column volumes in a 40–600 mM imidazole gradient in 50 mM Tris-HCl pH 8, 0.5 M NaCl. DTT was added to 5 mM to the pooled peak fractions that were further purified by SEC over a HiLoad Superdex 75 column 16/60 (GE Healthcare) equilibrated with 20 mM Tris-HCl pH 8, 500 mM NaCl, 2 mM DTT. Peak fractions were pooled, concentrated to 7 mg/ml and aliquoted before flash freezing in liquid nitrogen for storage at −80°C. We observed heavy protein precipitation even in a buffer containing 500 mM NaCl and therefore kept the protein concentration at 7 mg/ml or less. Untagged wild-type Aurora A expression and purification has been described previously (Janecek *et al.*, 2016).

### Production of TPX2 peptide

Recombinant peptide was produced using a method described elsewhere (Kuliopulos and Walsh, 1994). DNA encoding for residues 7–43 of human TPX2 (LSYSYDAPSD FINFSSLDDE GDTQNIDSWF EEKANLENLK GGGCQM) was cloned into pET31b expression vector (Novagen) and expressed in BL21(DE3) cells. 2 l of cell culture were grown in 2xYT media supplemented with 100 μg/ml ampicillin and 25 μg/l kanamycin at 37°C. Protein expression was induced at OD_600_ of 0.6 with 0.4 mM IPTG. Protein was expressed for 4 h at 37°C and the cells were harvested by centrifugation at 4000×*g* for 10 min at 4°C. Cell pellet was resuspended in water, lysed by three passages through an Emulsiflex C5 homogenizer and centrifuged at 30 000×*g*, 4°C for 30 min. Obtained inclusion bodies pellet containing fusion protein was dissolved in 20 mM Tris-HCl pH 8, 500 mM NaCl, 5 mM imidazole, 6 M guanidinium-HCl. Insoluble protein was removed by centrifugation 12 000 x g, 10 min at 4°C and supernatant was loaded onto 15 ml Ni-NTA beads (Qiagen). The column was washed with 150 ml of the loading buffer, and the protein was eluted with 20 mM Tris-HCl pH 8, 500 mM NaCl, 300 mM imidazole, 8M urea. The eluted protein was dialyzed overnight against 10 l water at 4°C and pelleted by centrifugation at 2000×*g*, 10 min, 4°C. 2 g of the pellet was solubilized in 60 ml of 80% formic acid, 2 g of CNBr was added and stirred for 20 h. The reaction mixture was evaporated to dryness, the protein was resuspended in 20 ml of 5% acetonitrile, 0.1% TFA and stirred for 1 h at room temperature. The suspension was centrifuged at 12 000×*g* for 10 min at 4°C and purified by reverse-phase HPLC on a 4.6 × 250 mm Vydac 5 μm 300 Å C18 reversed-phase column (HiChrom Ltd, UK) using 5–90% acetonitrile gradient. Fractions containing peptide were pooled and dried using centrifugal vacuum evaporator at room temperature followed by re-suspension in 10 mM HEPES pH 7.4, 100 mM NaCl.

### Labeling with Alexa Fluor 488

TPX2 peptide, TPX2 and CK2β display proteins containing free N-terminal cysteines were labeled with Alexa Fluor 488 (ThermoFisher Scietific). About 100 μg peptide or protein in 500 μl sodium phosphate pH 6.75, 150 mM NaCl were labeled using 30-fold excess of the maleimide dye. The reactions was allowed to proceed for 1 h at room temperature and the Alexa Fluor 488-maleimide was removed by dialysis using 3-kDa molecular weight cut-off membrane against 10 mM Tris pH 8, 250 mM NaCl buffer. Protein and peptide samples were dialyzed for 3 h at room temperature and further purified by SEC using Superdex 75 GL 10/300 or Superdex peptide 10/300 GL column (GE Healthcare). Labeled peptides and proteins were concentrated to 100 μM, flash frozen in liquid nitrogen and stored at −80°C. The concentration of labeled peptide or proteins and degree of labeling was determined by measuring the absorbance at 280 and 495 nm.

### Sedimentation velocity analytical ultracentrifugation

Protein samples were prepared for SV AUC experiments by buffer exchanging of concentrated protein stocks into 20 mM HEPES pH 7.5, 500 mM NaCl. CK2α kinase and RAD-CK2β display were loaded in cell assemblies at 6 and 14.3 μM concentration, respectively, and the complex was formed by mixing 5 μM of each of the two proteins together. Aurora A_D274N_ and RAD-TPX2 were loaded in cell assemblies at 10 and 25 μM concentration, respectively, and the complex formed by mixing the two proteins at 10 μM each.

SV AUC experiments with absorbance detection were performed in an Optima XL-1 analytical centrifuge at 50 000 rpm at 20°C after 60 min equilibration step. A total of 100 scans were acquired at 280 nm. Data were analyzed using the SEDFIT software (Brown and Schuck, 2008) and the continuous size-distribution option. Buffer density and viscosity at 20°C were calculated using SEDNTERP.

### Analytical SEC

Purified protein samples, either as individual proteins or equimolar mixtures, were separated by analytical SEC over an Superdex 75 5/150 size exclusion column (GE Healthcare) equilibrated with 20 mM HEPES pH 7.5, 500 mM NaCl. Fractions from the peaks from each run were analyzed by SDS-PAGE with purified proteins as standards. The column was calibrated using molecular weight standards (blue dextran, bovine serum albumin, carbonic anhydrase, cytochrome C and aprotonin, Sigma-Aldrich cat. MWGF70) and the calibration curve used to estimate the molecular weights of the three samples (Fig. S5).

### Isothermal titration calorimetry

All ITC experiments were performed on itc200 instrument (GE Healthcare). Prior to ITC titration, proteins and peptides were buffer exchanged into 10 mM HEPES pH 7.4, 500 mM NaCl, 0.05% P20 surfactant. ITC experiments with Aurora A_wt_ kinase were performed in buffer containing 50 mM HEPES pH 7.4, 100 mM Mg(CH_3_COO)_2_, 100 mM NaCl, 1 mM ATP, 1 mM DTT, 0.01% v/v P20. Titrations experiments were conducted at 25°C with an initial 0.4 μl injection at a duration of 0.8 s, followed by 19 or 29 injections of 2 μl at a duration of 4 s with 120, 180 or 200 s spacing for Aurora A_D274N_, Aurora A_WT_ and CK2α kinase binding assays, respectively. In the Aurora A_D274N_ and Aurora A_WT_ binding assays, 50 μM solution of the titrant was injected into 5 μM kinase solution in the cell. For the determination of binding affinity between CK2α and its interacting partners, 100 μM of CK2β peptide or RAD-CK2β display were titrated into 10 μM CK2α kinase solution. Binding isotherms were fit by non-linear regression using the single-site model provided by ORIGIN software (Origin Lab). The stoichiometry of the interaction (N), equilibrium association constant (K_A_) and change of enthalpy (ΔH) were floated during the fitting.

### Surface plasmon resonance

SPR analyses were conducted using a Biacore T100 instrument (GE Healthcare) using CM5 sensor chips to which cRAD display proteins were immobilized through their unique cysteine residues. All coupling reagents were purchased from GE Healthcare: N-ethyl-N′-dimethylaminopropylcarbodiimide (EDC), 2-(2-pyridinyldithio) ethaneamine hydrochloride (PDEA), N-hydroxysuccinimide (NHS). All immobilizations were conducted at 25°C in running buffer 10 mM HEPES pH 7.4, 500 mM sodium chloride, 0.05% P20. Two CM5 sensor chips were used for the experiments. Peptides and RAD scaffold displaying the peptides were covalently immobilized via free cysteine on the sensor surface in cells 2 and 4, respectively. Cells 1 and 3 were derivatized with cysteine only and served as reference surfaces. Active and reference cells were created automatically using the immobilization surface preparation Wizard software. Immobilization levels for cells 2 and 4 ranged from 240 to 400 RU. For binding studies, 2.5 μM protein stocks of CK2α and Aurora A_D274N_ were prepared in running buffer from which twofold serial dilutions were made spanning concentrations from 2.5 nM to 2.5 μM. Each concentration was injected at 10 μl/min for 1400 s (CK2α) and 1500 s (Aurora A_D274N_) across all four cells. The dissociation step was 180 s after which sensor surfaces were regenerated by injecting 3 M MgCl_2_ solution 3 times for 30 sat 60 μl/min. All sensograms were processed using double-referencing method followed by dissociation constant determination using 1:1 steady-state affinity model implemented into the BIAevaluation 3.0 software. To minimize the effect of unspecific binding, the CK2α data was analyzed at 200 s post-injection. Aurora A binding to TPX-2 peptide and RAD-TPX-2 were analyzed also using kinetic data with heterogenous ligand model, with data presented in the Supplementary Material.

### Fluorescence anisotropy

A stock solution of Alexa Fluor 488-labeled peptide or display proteins was diluted in assay buffer to concentration of 20 nM. For both studies, 10 mM HEPES pH 7.4, 500 mM NaCl, 1 mM DTT, 0.05% P20 buffer from was used. Protein concentrations ranged from 2 nM to 2 μM for CK2α and 2 nM to 1.5 μM for Aurora A_D274N_. Samples were prepared in a black polysterene, flat-bottomed, 96-well half-area plate (Corning Inc.) and incubated for 15 min at room temperature prior measurements. FA was measured using PheraStar spectrofluorimeter plate reader (BMG Laboratories, Durham, NC) equipped with polarization filters (excitation 485 nm, emission 520 nm). Dissociation constants were calculated in pro Fit (Quantum Soft) by fitting experimental data to 1:1 binding model equation.

### FA competition assay

FA binding and competition experiments were performed at 25°C as described above in 50 M HEPES pH 7.4, 0.1 M Mg(CH_3_COO)_2_, 100 mM NaCl, 1 mM ATP, 1 mM DTT, 0.01% v/v P20.

In order to perform competition experiments, the affinity of the Alexa Fluor 488-labeled TPX-2 peptide for the kinase domain of Aurora A_WT_ was determined by titrating increasing concentration of the kinase into constant 10 nM concentration of labeled TPX2 peptide. Direct binding measurements were performed using a constant peptide concentration. The concentration of protein titrant was adjusted based on the dissociation constant observed in trial experiments. Binding isotherms were fitted to a standard quadratic equation for a single-site.

Competitive binding experiments were performed using 10 nM Alexa Fluor 488-labeled TPX2 peptide and 50 nM Aurora A_WT_, determined to give ~90% saturation of binding in the absence of a competitor. The interaction between the protein and the TPX2 peptide was then challenged using eight different variants of the RAD-TPX2 protein as competitors. Serial dilutions of each competitor were mixed with Aurora A and labeled TPX-2 peptide with the final concentrations of the competitor ranging from 195 nM to 25 μM. *K*_i_ for individual mutants was obtained from fitting of experimental competitive binding isotherms into the equation derived by Wang (1995).

### Bio-Layer Interferometry

Octet RED96 (ForteBio) instrument was used for BLI analysis for double His-tagged RAD-TPX2/Aurora A_WT_ interactions. Experiments were performed using Anti-Penta-HIS biosensors (ForteBio), which were regenerated with 10 mM glycine, pH 1.7. IMAC purified RAD proteins displaying wild-type sequence or alanine mutants of the TPX2 were buffer exchanged into 50 mM HEPES pH 7.4, 500 mM NaCl, 100 mM Mg(CH_3_COO)_2_, 0.5 mM ATP, 0.5 mM TCEP, 0.05% P20 (assay buffer). For binding assays pre-wet sensors were loaded with double His-tag labeled proteins at 1 μM in assay buffer. Dilution series (from 0 to 500 or 5000 nM) of Aurora A_WT_ kinase construct without His-tag was prepared in the assay buffer and following experimental setup was used for each measurement cycle: regeneration 3 × 5 s, baseline monitoring 120 s, sample loading 200 s, baseline monitoring 150 s, ligand binding (association) 400 s and ligand dissociation 600 s, followed by regeneration and the next cycle. Regenerated Anti-Penta-HIS biosensors were used to perform double referencing. Data was processed using ForteBio Analysis 7.1 software and dissociation constants were calculated in pro Fit (Quantum Soft) by fitting experimental data to 1:1 binding model equation.

## Supplementary Material

Supplementary Data
