# Supplementary material for: Development of a multipurpose scaffold for the display of peptide loops
Source: Protein Eng Des Sel. 2017 Apr 24;30(6):419–30. doi: 10.1093/protein/gzx017 (PMC5897841; doi:10.1093/protein/gzx017)
Supplement: Supplementary Data [file rossmann_et_al_supplementary_information.pdf]

Supplementary Information

Rossmann et al. Multipurpose System for Displaying of Peptide Loops

Contents

Figure S1. Vector maps 2

Figure S2. Structures of Aurora A and CK2α kinases. 3

Figure S3. Overview of LIC cloning 4

Figure S4. Analytical ultracentrifugation data 5

Figure S5. MW estimation of CK2α, RAD-CK2β$_{(186\text{-}200)}$ and their complexes by SEC 6

Figure S6. Mass spectrometric analysis of peptides 7

Figure S7. ITC data. 8

Figure S8. Thermodynamic parameters of binding 9

Figure S9. SPR sensograms 10

Figure S10. Purification of RAD-TPX2 mutants. 12

Figure S11. BLI sensograms. 13

Figure S12. Determination of binding affinity between TPX2 and Aurora A$_{WT}$ 14

Figure S13. Expression of finger epitopes of TGF-β growth factors. 15

Table S1. Summary of current monomeric scaffold technology 16

Table S2. Oligonucleotides 18

Table S3. Proteins used in this study. 19

Supplemental References: 19

Figure S1. Vector maps

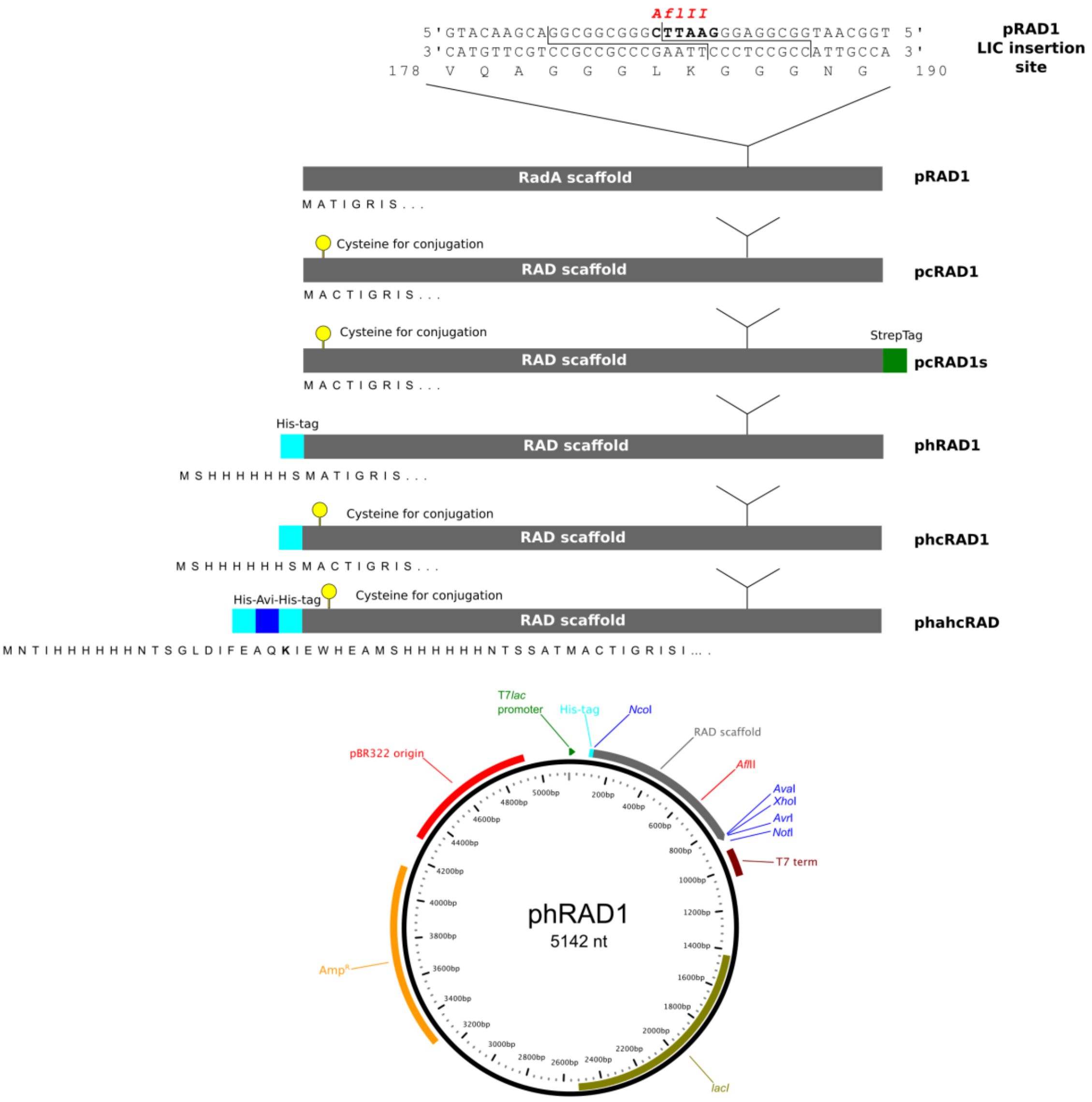

Vector	Molecular weight (Da)	Isoelectric point	Molar Exctintion coefficient at 280 nm ($M^{-1}cm^{-1}$)
pRAD1	25942.64	7.65	11460
pcRAD1	26045.78	7.59	11460
phRAD1	27070.83	7.81	11460
phcRAD1	27173.97	7.74	11460

Figure S1. Vector map for various pRAD vectors. The position for inserting displayed peptide encoding sequences is shown on the top with its LIC cloning sequence. Sequences of N-terminal tags and the start of the RAD scaffold sequence are shown for each variant. The table below provides some of the key biochemical properties of the empty RAD scaffold variants.

Figure S2. Structures of Aurora A and CK2α kinases.

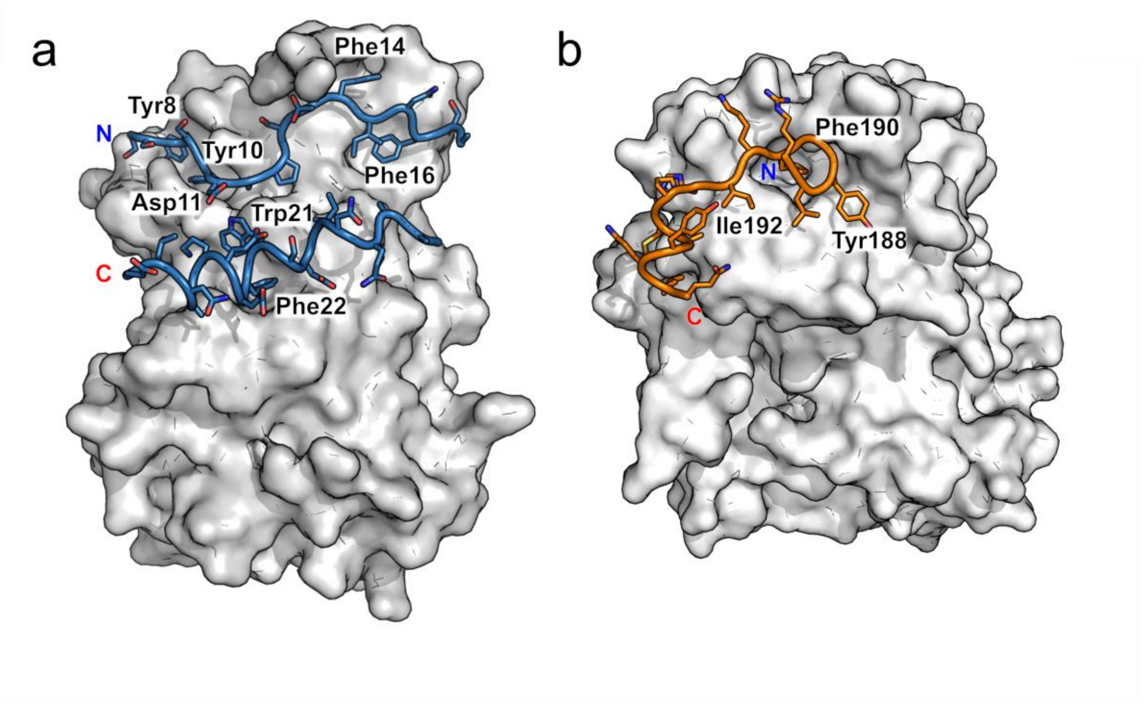

Figure S2. Structures of Aurora A and CK2α in complex with their binding partners. (a). Aurora A kinase domain (white surface) complexed with peptide corresponding to residues 2-43 of TPX-2 (blue), with side chains shown as sticks and key residues labelled (PDB: 3e5a). (b). Human CK2α kinase (white surface) with key binding epitope as used in this work, from the regulatory subunit CK2β (orange) with side chains shown as sticks (PDB: 4dgl).

Figure S3. Overview of LIC cloning

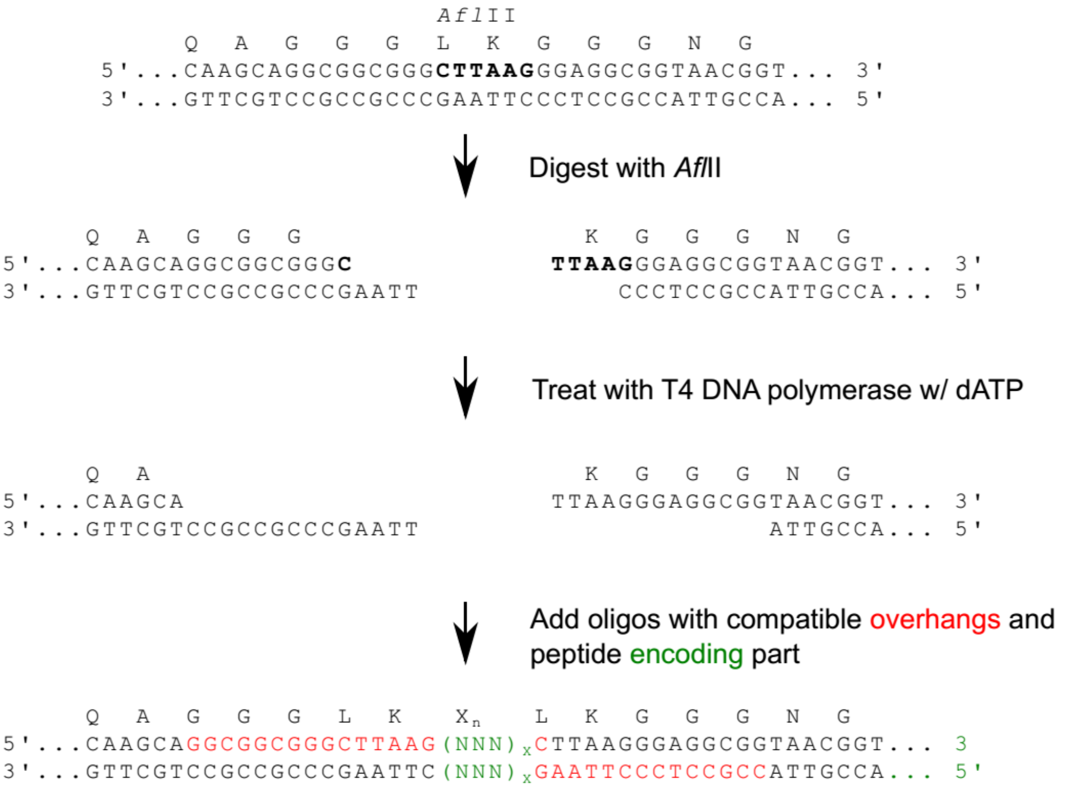

Figure S3. Overview of the ligation independent cloning (LIC) protocol used for inserting epitope encoding sequences into the pRAD plasmids.

Figure S4. Analytical ultracentrifugation data

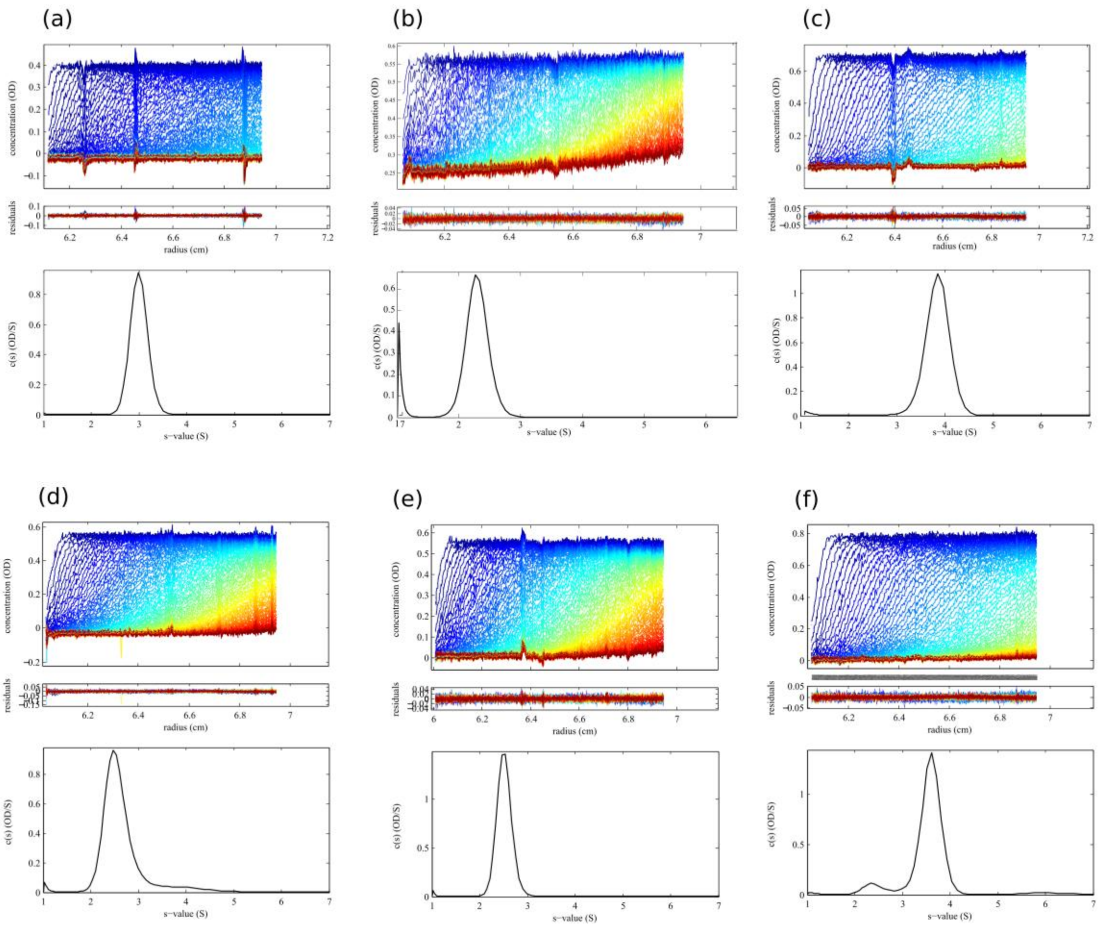

Figure S4. Sedimentation velocity AUC data for Aurora A_{D274N} and CK2α kinases, RAD displays and kinase/RAD display complexes. (a) Human CK2α kinase (b) RAD-CK2β (c) RAD-CK2β/CK2α kinase complex. (d) Aurora A_{D274N} kinase (e) RAD-TPX2 (f) RAD-TPX2/Aurora A_{D274N} kinase complex. In each data set, the top panel shows the absorbance data and the fits, the middle panel depicts the residuals of the fits to the data and bottom panel shows the *c*(s) distribution of the species fit to the data from each run.

Figure S5. MW estimation of CK2α, RAD-CK2β$_{(186-200)}$ and their complexes by SEC

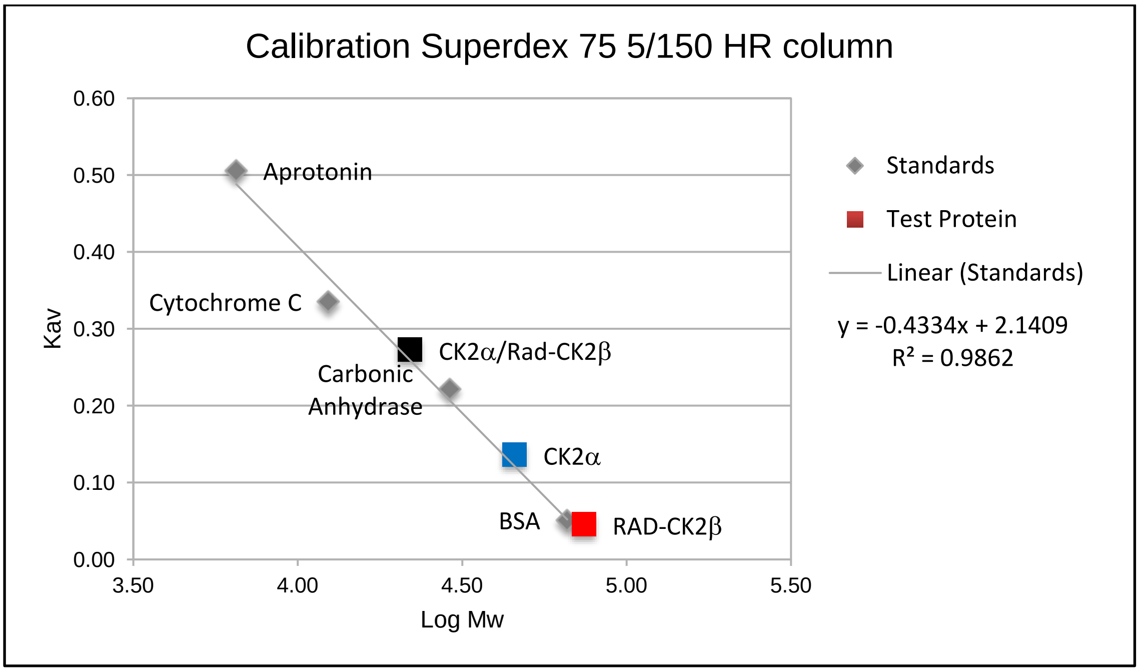

Figure S5. Molecular weight protein standards (Sigma-Aldrich, cat. no. MWGF70, gray diamonds in the plot) were used to calibrate Superdex 75 5/150 column. Blue dextran was used to define the void volume (1.24 ml)and total volume was assumed to be 5 ml. Elution positions of CK2α,(blue square) RAD-CK2β$_{(186-200)}$(red square) and their complex (black square) , as shown in Fig 2(c) in the main article, were used to estimate the molecular weights of the proteins from the calibration curve.

Figure S6. Mass spectrometric analysis of peptides

(a)

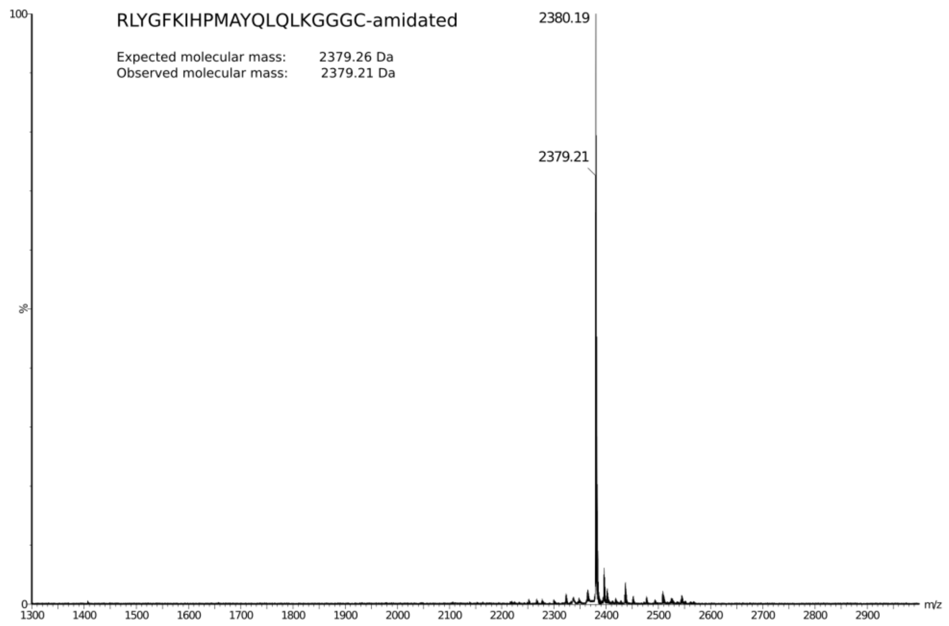

(b)

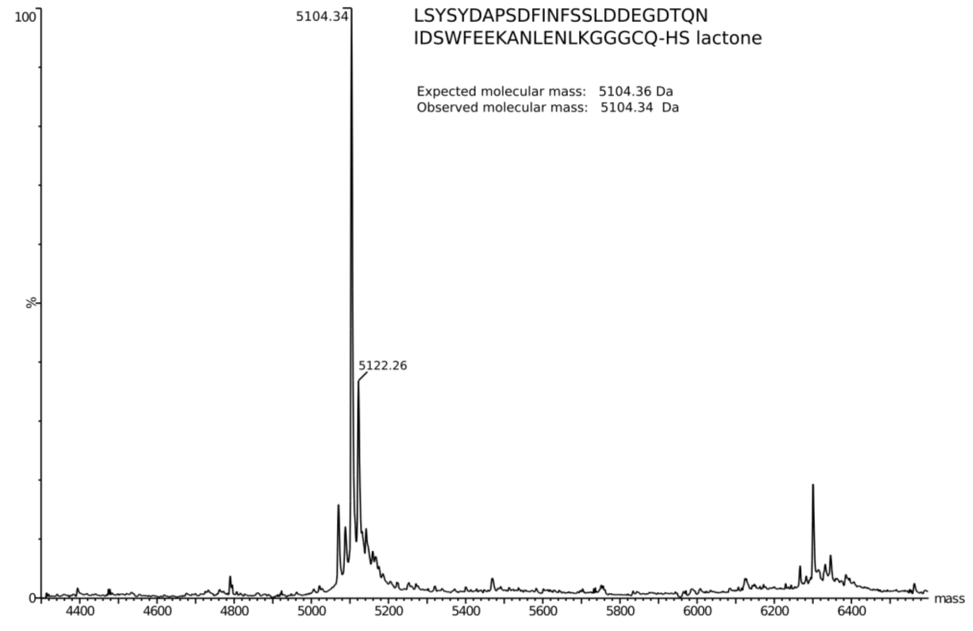

Figure S6. ESI analysis of the peptides used in this work. (a) Purified synthetic linear CK2β peptide. (b) Purified TPX2 peptide released from KSI fusion through cyanogen bromide cleavage, with homoserine lactone as the terminal residue after the cleavage.

Figure S7. ITC data.

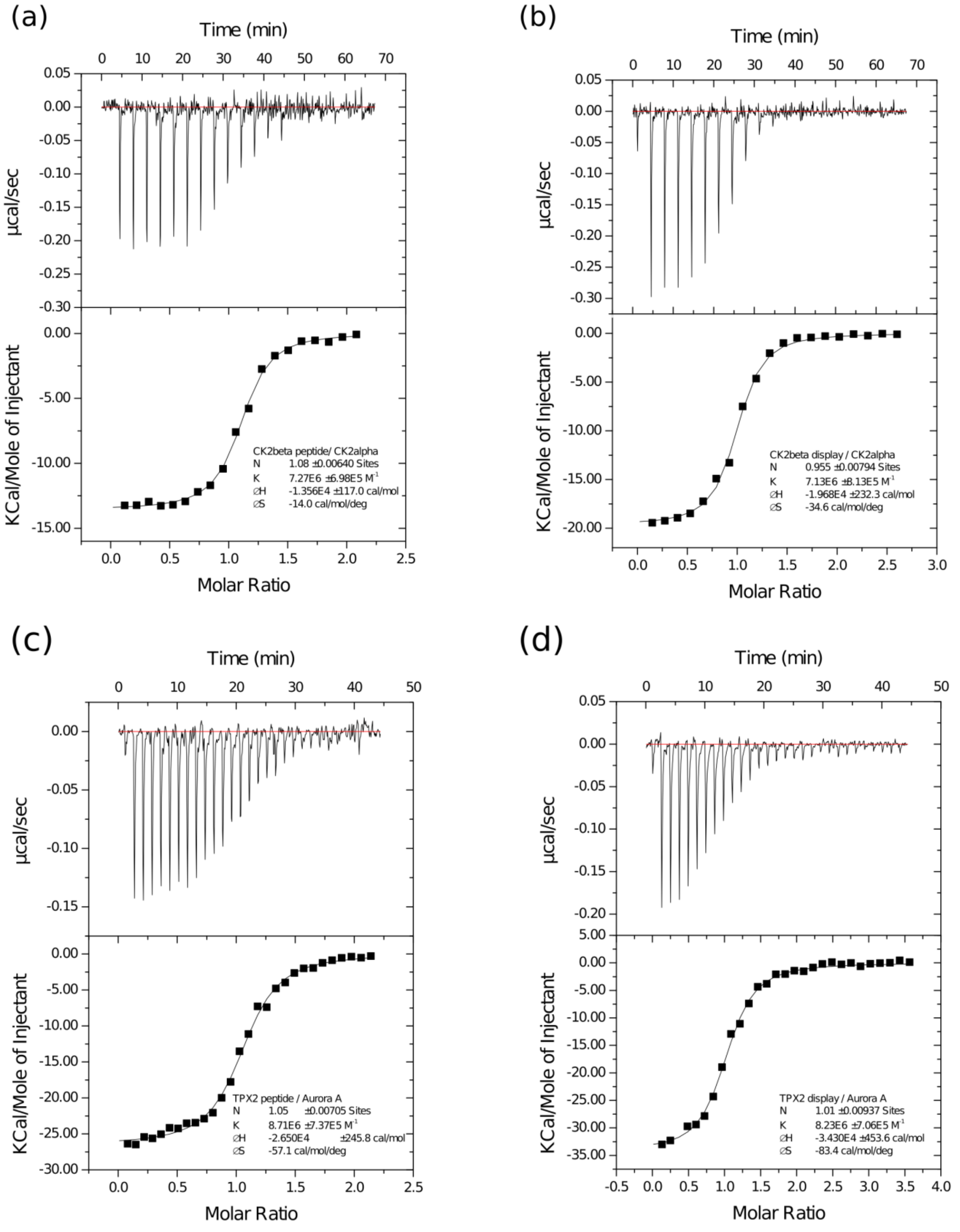

Figure S7. ITC traces of the binding interaction of the CK2α (a, b) and Aurora A_{D274N} (c, d) kinases to their peptide binding partners provided as either linear peptides (a, c) or displayed by the RAD scaffold (b, d).

Figure S8. Thermodynamic parameters of binding

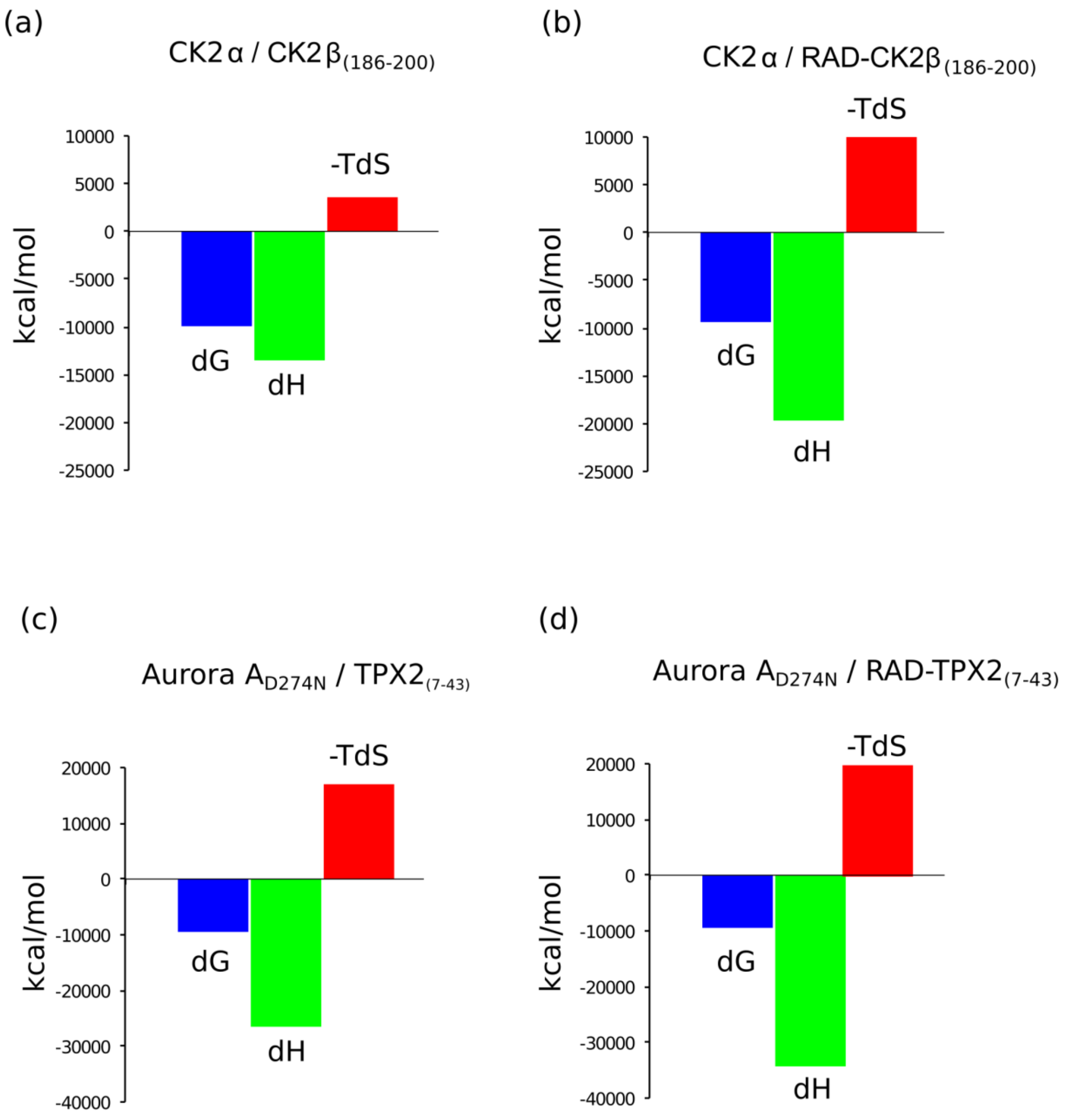

Figure S8. Thermodynamic parameters of kinase-peptide interaction as determined by ITC. Binding interaction of CK2α (a, b) and Aurora A_{D274N} (c, d) kinases to their peptide binding partners provided as either linear peptides (a, b) or displayed by the RAD scaffold (b, d).

Figure S9. SPR sensograms.

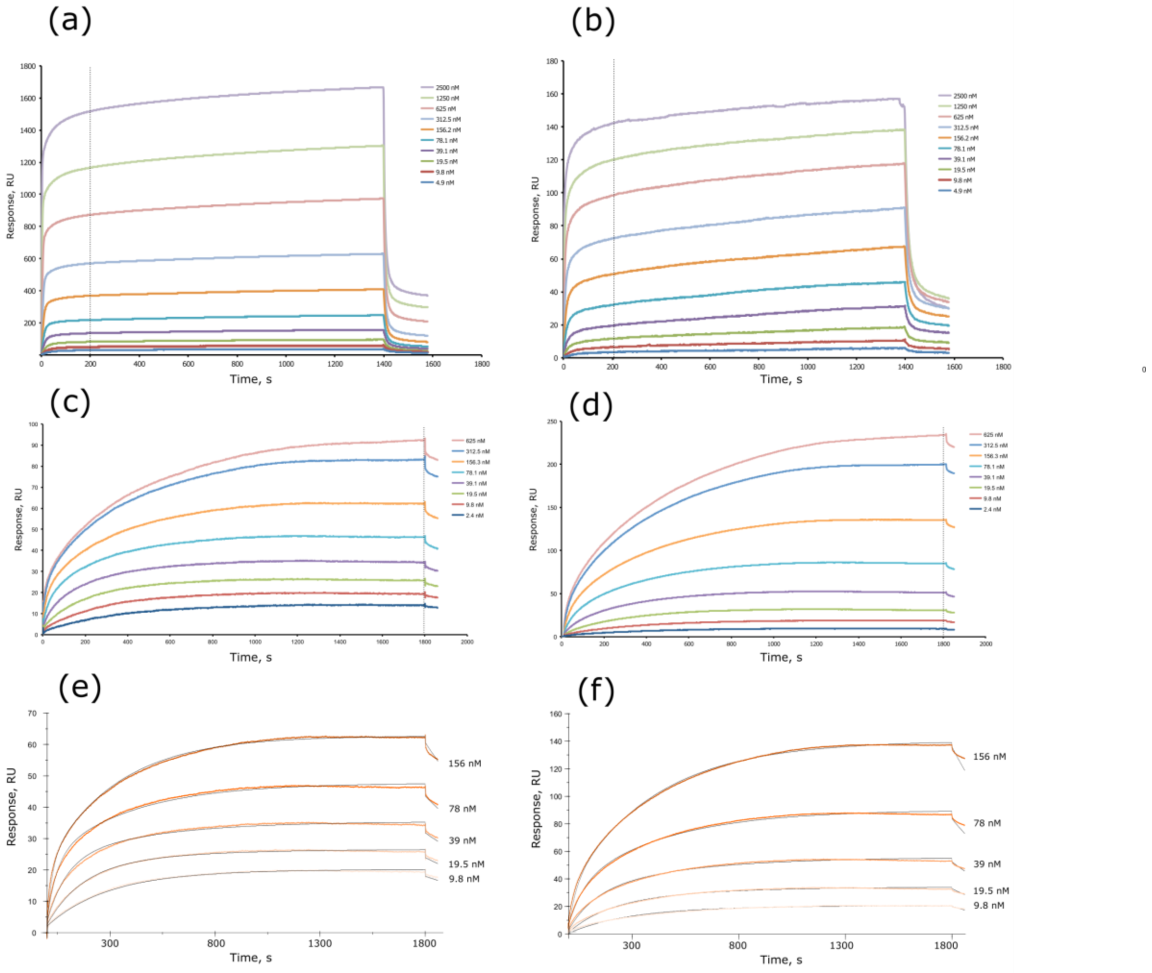

Figure S9. SPR sensograms of the analysis of the interactions of CK2α (a and b) and Aurora A D274N (c and d) kinases with their binding partners presented in of linear peptide form or as peptides grafted onto the RAD display system. (a) CK2α/CK2β peptide. (b) CK2α/RAD-CK2β. (c) Aurora A $_{D274N}$ /TPX2 peptide. (d) Aurora A$_{D274N}$/RAD-TPX2$_{7\text{-}43}$. Dotted vertical line indicates the time point from which data was used in the equilibrium analysis. (e) Aurora A $_{D274N}$ /TPX2 peptide SPR data analysed using kinetic data (fit shown as black line). (f) Aurora A$_{D274N}$/RAD-TPX2$_{7\text{-}43}$ SPR data analysed using kinetic data (fit as black line).

Due to heterogeneity in both of the ligands (TPX2$_{7\text{-}43}$ and RAD-TPX2$_{7\text{-}43}$) and in the analyte (Aurora A$_{D274N}$), the SPR data displays some non-ideal behaviour. This is characterised by multiphasic and slow kinetics. The data were recorded expecting a fast equilibration and thus that an instantaneous dissociation would occur, but the curves show that this was clearly not the case, and so the actual dissociation phase was unfortunately not recorded beyond 60 seconds.

When fitting to multiphasic kinetic models, a heterogeneous ligand model provides the best fit (black line in the graphs). This may be explained by the long TPX2$_{7\text{-}43}$ peptide and RAD displayed loop existing in different conformations that show different kinetic binding behaviour. In the absence of further evidence for this, and to avoid over-parameterisation of the fit, we have used equilibrium binding experiments to compare the interaction of the TPX2 peptide with the scaffold-displayed version. This additionally provides a more direct comparison to the

other equilibrium experiments we have used to compare the interaction of Aurora A_{D274N} with RAD displayed or linear peptide. Simulations (Biasimulation software) and empirical experiments defined the optimal injection time (1800s) to allow the system to approach equilibrium while preserving the solubility/functionality of Aurora A_{D274N}. This allowed us to define reasonably accurate upper limits for the equilibrium dissociation constants for these two interactions, despite the observed non-ideality in both datasets.

Dissociation contants for Aurora A:TPX interaction from the kinetic analysis of the SPR data using heterologous ligand model:

	K_{D1} (nM)	K_{D2} (nM)
$TPX2_{7-43}$	327	4.1
RAD-$TPX2_{7-43}$	1,043	15.4

Figure S10. Purification of RAD-TPX2 mutants.

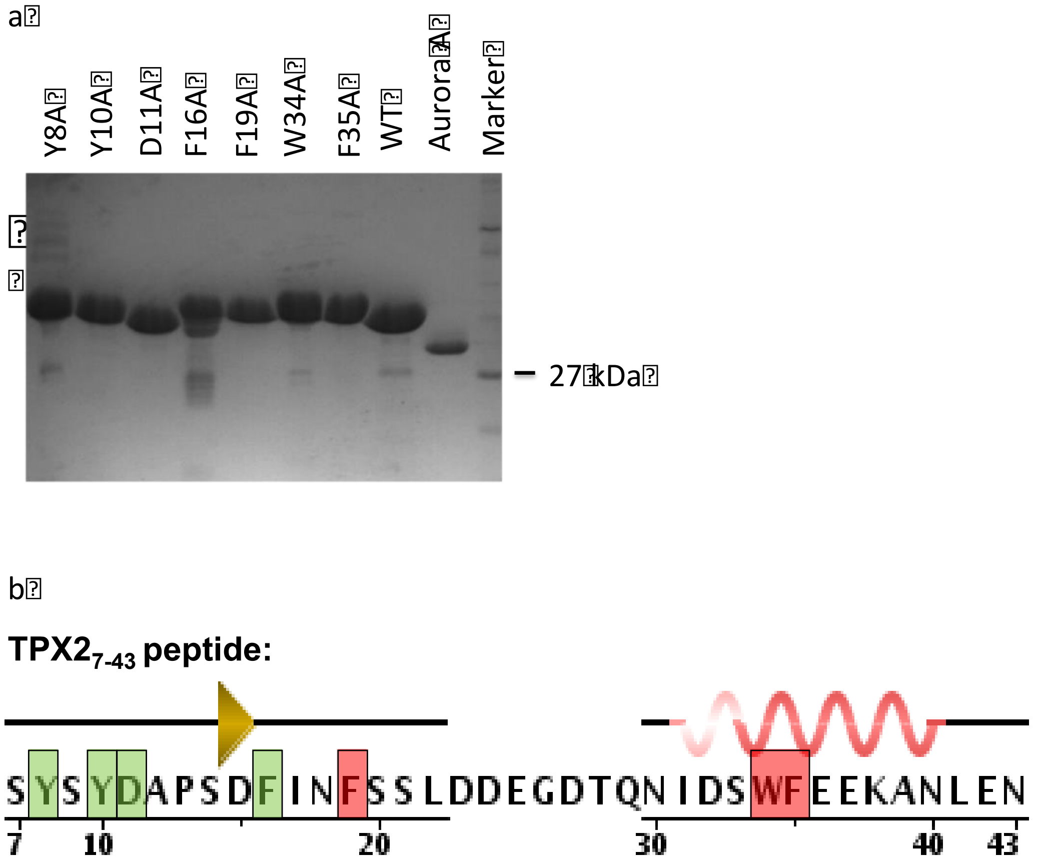

Figure S10. (a) SDS-PAGE of purified Aurora A$_{WT,}$ RAD-TPX2 and its alanine mutants, as labelled on the top of the gel. (b) Representation of the primary and secondary structure of the TPX2 peptide when bound to Aurora A. Residues mutated to alanines are highlighted by coloured boxes.

Figure S11. BLI sensograms.

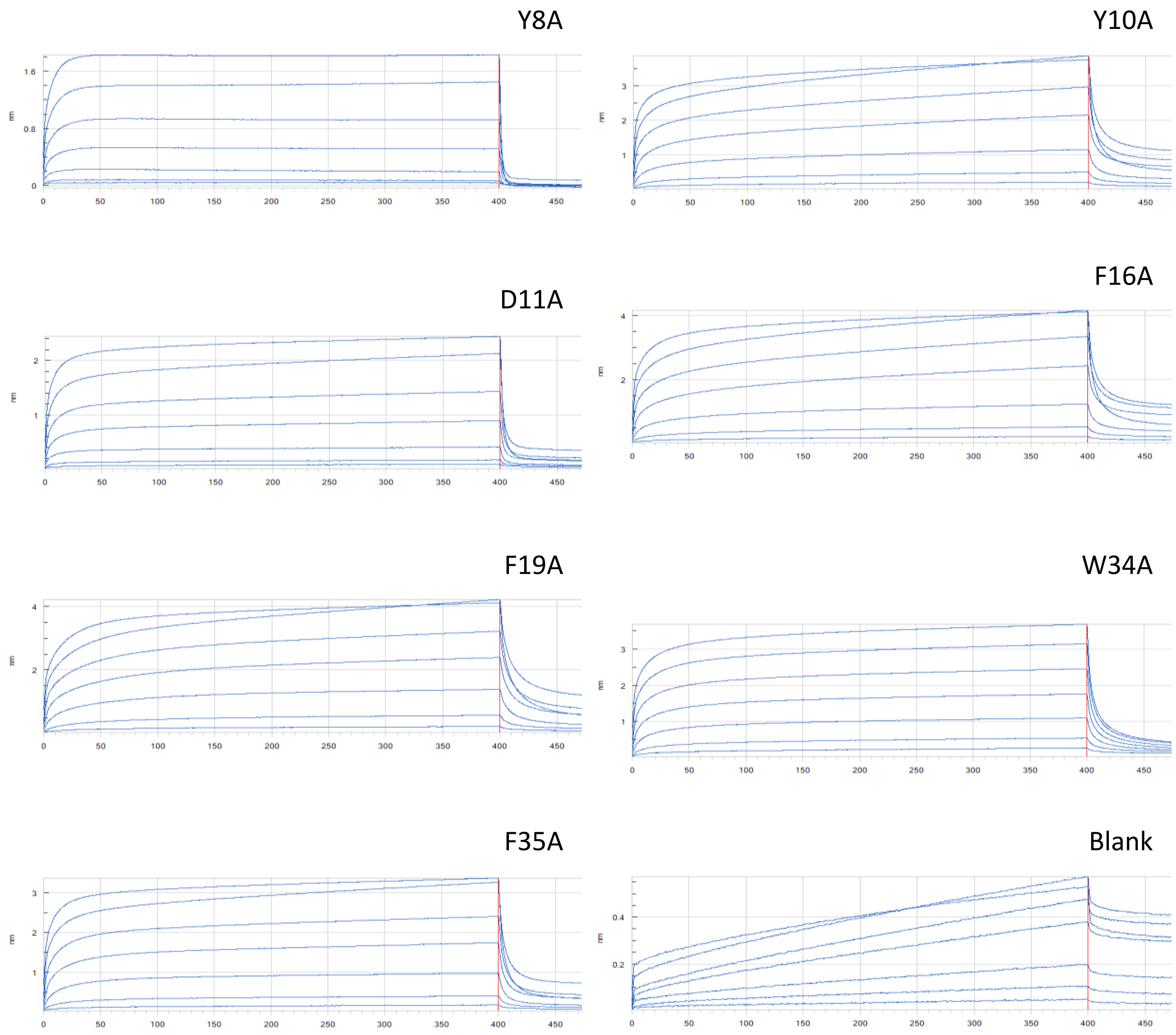

Figure S11. BLI sensograms for the analysis of the interactions between Aurora A_{WT} kinase and RAD-TPX2 alanine mutants.

Figure S12. Determination of binding affinity between TPX2 and Aurora A_{WT}

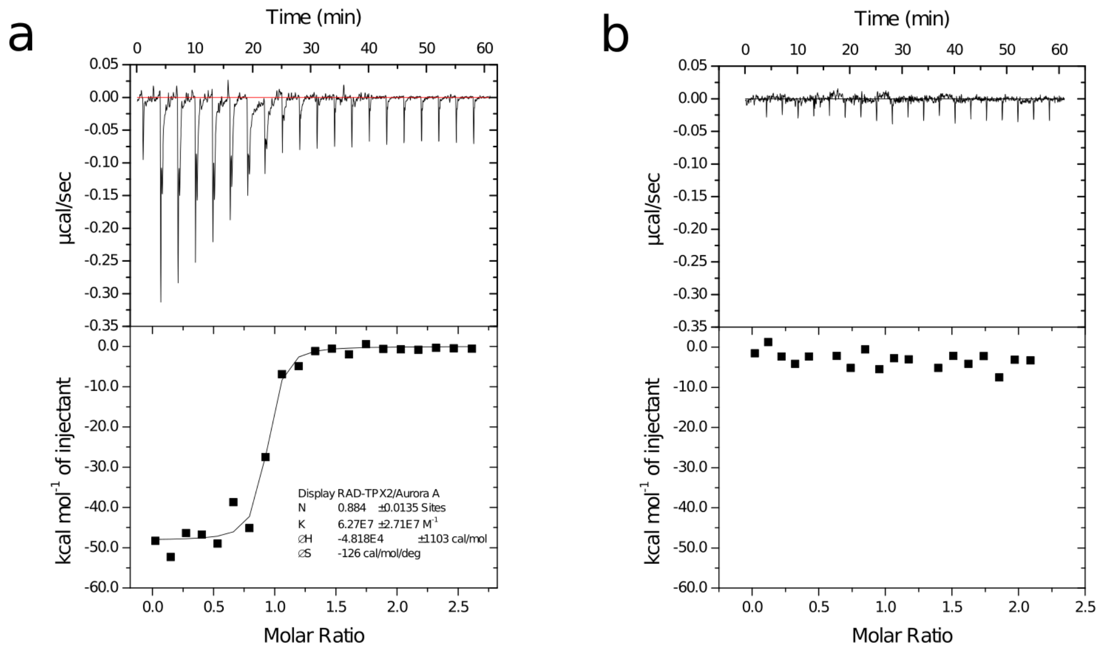

Figure S12. (a) ITC data for cRAD-TPX2 and Aurora A_{WT} interaction showing a K_D of 15.9 nM. (b) Titration of empty RAD scaffold into Aurora A_{WT} showing only heats of dilution and no unspecific interactions between the scaffold and the kinase.

Figure S13. Expression of finger epitopes of TGF-β growth factors.

a

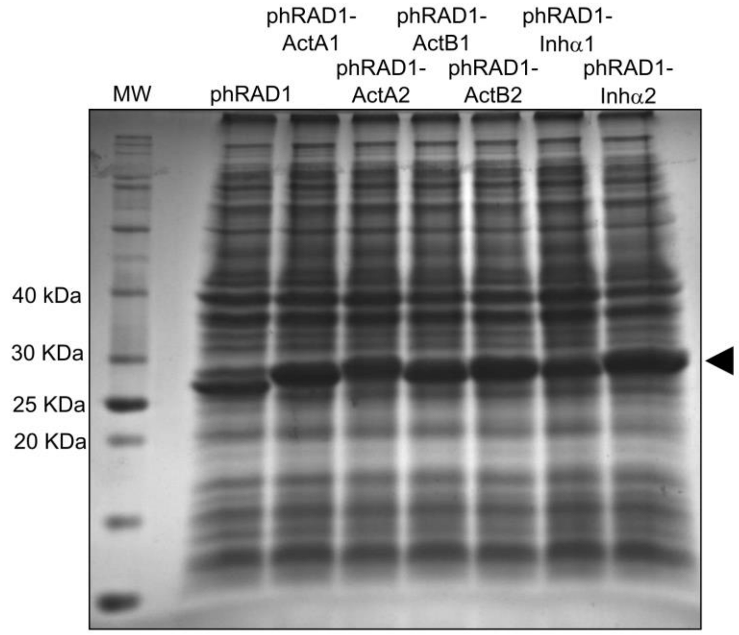

b

Activin A Finger 1 GGGLK**QFFVSFKDIGWNDWIIAPSGYHAN**LKGGG
Activin A Finger 2 GGGLK**KLRPMSMLYYDDGQNIIKKDIQNMIVE**LKGGG
Activin B Finger 1 GGGLK**QFFIDFRLIGWNDWIIAPTGYYGN**LKGGG
Activin B Finger 2 GGGLK**KLSTMSMLYFDDEYNIVKRDVPNMIVE**LKGGG
Inhibin α Finger 1 GGGLK**ALNISFQELGWERWIVYPPSFIFH**LKGGG
Inhibin α Finger 2 GGGLK**PGTMRPLHVRTTSGGYSFKYETVPNLLTQ**LKGGG

c

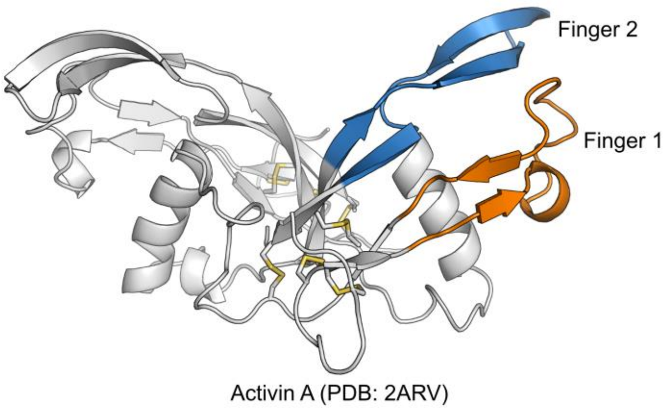

Figure S13. Expression of finger epitopes from TGF-β growth factors in phRAD1. (a) SDS-PAGE analysis of soluble fractions of E. coli lysates from cells expression finger 1 and figner 2 epitopes from activin A (ActA), activin B (ActB) and inhibin α (Inhα) with empty phRAD1 as control in the first lane. (b) Sequences of the each of the finger epitope sequences (in bold) with the linker residues from the RAD display shown at both ends. (c) Structure of mature activin A with fingers 1 and 2 coloured orange and blue, respectively.

Table S1. Summary of current monomeric scaffold technology

Name	Class	Structure	Protein	Source	Residues/ crosslinks	Display residues	Target	Tm (°C) Method	Best K_D (M)	Company	Refs
**DNA BINDING PROTEINS**											
**RAD**	2 Loops	4A6P α/β	RadA	Archea	238/0	52 in loop	CK2α, AuroraA	95 DSF	10^{-7}	-	This work
**Affitin/ Nanofitin**	Surface	4CJ2 5 strand β- barrel	Sac-7d	Archea	66/0	14 on β-sheet, 2 loops	PulD GFP IgG	>100 Autoclave	10^{-9}	Affilogic	(1–3)
**PROTEASE INHIBITORS**											
**Adhiron**	2 loops	4N6T 4 AP β, central α1	Phytocystatin protein	Plant	100/0	9 in loops 1 & 2.	Yeast SUMO, Magnetite	~87 DSC	~ 10^{-8}	-	(4, 5)
**Affimer**	2 loops, N-terminus	1NB5 4 AP β, central α1	Stefin A/Cystatin	Human	98/0	10-42 in loops 1,2 or N-term	CDK2, Mcl-1, POZ of BCL6, Bcl-2	~80 CD	~ 10^{-9}	Avacta Life Sciences	(6–8)
**Kunitz domain**	1-2 loops	1ZR0 α/β	BPTI, APPI, HPSTI	Human	58/3	5-8 in loops	Neutraphil elastase, TF-FVIIIa Kallikrein	-	10^{-12}	DYAX	(9–13)
**CELL SURFACE PROTEINS**											
**ABD**	Surface	1GJT α3	Streptococcal Protein G Albumin binding domain	Bacterial	46/0	15 surface on two helices	PSP94, TNFα, HER3	60 DSF	10^{-8}	-	(14–16)
**Affibody**	Surface	1LP1 α3	Staphylococcal Protein A Z domain	Bacterial	58/0	13 surface on two helices	EGRF, Her2, TNFα, etc	60-75 CD	5×10^{-15}	Affibody AB	(17–20)
**Adnectin/ Monobody**	3 Loops only, 3 Loops & side of β - sheet	3RZW, 3UYO β sandwich of 7 β-sheets	10th domain Fibronectin III	Human	94/0	1-9 in 3 loops, 4 in 2 loops & 2 β - strands	VEGF-R2, EGRF, IGRF	53-73 CD	10^{-9}-10^{-8}	Adnexus Therapeutics	Reviewed in (21) (22–24)
**Pronectin**	Loops	3R8Q 2 β -sheets	14th domain Fibronectin III	Human	90-95/0	6-15 in 3 loops	VEGF-R2, AXL,	-	<10^{-7}	Protelica	(25)

**Centyrin**	Surface	3TES<br>β-sheet	hTenascin C	Human	89/0	13 in loops & strands	Frizzled receptor<br>c-MET	45-87<br>DSC	10^{-10}	-	(26)
**STRUCTURAL PROTEINS**											
**-**	2 variable loops	1HA4<br>2 β-sheet	γ-S-crystallin CTD	Human	86/0	12-13 in loops	HIV integrase	-	10^{-7} (K_i)	-	(27)
**Affilin**	Surface	2JDG<br>β-sheet	γ-B-crystallin	Human	176/0	8 on β-sheet	HPV E7<br>proNGF<br>Estradiol	56-72<br>DSC	10^{-8}	SCIL Proteins	(28, 29)
**Affilin**	Surface	1UBI<br>α/β	Ubiqutin	Human	76/0	6 on β-sheet/loop	TNF-α<br>ED-B	76<br>CD	10^{-11}	SCIL Proteins	(30, 31)
**ENZYMES/LIGAND BINDING PROTEINS**											
**Anticalin**	4 variable loops	1BBP,<br>4GH7,<br>1N0S<br>8 stranded β- barrel	Bilin-binding protein (BBP),<br>Apolipoprotein D, Lipocalin 1 & 2	Human, Insect	160-180/<br>0-2 S-S	4 loops (up to 24 in each)	HGRF, VEGF-A, CTLA-4, small molecules	60-70<br>CD	$<10^{-9}$	Pieris AG	Reviewed in (32)<br>(33)
**Fynomer**	2 loops	4AFQ<br>β -sandwich	Fyn Tyrosine kinase	Human	63/0	6 in 2 loops	Abl-1,<br>Src,<br>chymase	WT: 70<br>DSC	10^{-10}	Covagen	(34–36)
**Obody/ OB fold**	Surface and loop	4GLA<br>5 strand<br>β- barrel	Aspartyl<br>tRNA synthetase	Archea	111/0	17-24 in loop &<br>β- strand	Egg white lysosyme	65-80<br>DSC	10^{-9}	-	(37)
**PROTEASES AND TOXINS**											
**Knottins**	Loops	1HYK,<br>2IT7,<br>2-3 β strands & cysteine knot	CBD, AgRP EETI-II,<br>Min-23,<br>Scorpion toxin	Plant, invertebrat es	23-37/3	4-8 in loops/turns	Chymotrypsin, trypsin, integrins, amylase	-	10^{-10}	Selecore	(38–40)
**DESIGNED PROTEIN**											
**Alphabody**	Surface	4OE8<br>3 AP α<br>coiled coil	-	Designed	70-100/0	11 surface on helices A and C	IL-23	~120<br>CD + GnHCl	10^{-9}	Complix	(41)

Table S2. Oligonucleotides

Oligo name	Site	Oligo sequence
		**Cloning Primers**
**CK2α3 F**	*BspH*I	5′ AATTCTCATGAGCGGACCCGTGCCAAGCAGG
**CK2α4 R**	*BamH*I	5′ GGGGGATCCTCAACCCATTCGAGCCTGGTCCTTCAC
**pRAD1**	*Nco* I	5' ATATATCCATGGCTACAATAGGTCGTATTTCCACGG
**HAT2**	none	5′ TATATGTTTAAACCCCTCAAGACCCGTTTAGAGG
**pcRAD1**	*Nco* I	5' GACCCATGGCTTGCACAATAGGTCGTATTTCC
**pRAD-TPX2-F**	*Nco* I	5' TATATAGGATCC**ATG**GCTTGCACAATAGGTCG
**pRAD-TPX2-F**	*Not* I	5' TATATAGCGGCCGCCTCGAGTTAATCCTCTATCCCTTTTTCAG
**phRAD-TPX2-F**	*BspH*I	5' TAATAATC**ATG**AGCCATCACCATCACCATCACAACAC
**TPX2 F4**	*AlnWI*	5′ AACAGATGCTGAGCTATAGCTATGATGCGCCGAGC
**TPX2 R4**	*AlnWI*	5′ AACAGCATCTGGCAACCGCCTCCCTTCAGGTTTTCCAGGTTCGCTTTTTCTTCAAACC
		**Mutagenesis Primers**
**CK2α5 F**	n/a	5′ CCCATAATGTTATGATTGATCACGAGCACAGAAAGCTACGAC
**CK2α6 R**	n/a	5′ GTCGTAGCTTTCTGTGCTCGTGATCAATCATAACATTATGGG
**pRAD-LIC1**	*Afl* II	5' CTAACCAAGTACAAGCA**GGCGGCGGGCTTAAGGGAGGCGGT**AACGGTCACATCTTGGCCC
**pRAD-LIC2**	*Afl* II	5' GGCCAAGATGTGACCGTT**ACCGCCTCCCTTAAGCCCGCCGCC**TGCTTGTACTTGGTTAG
**RAD-K144A-F**	n/a	5′ CGGTAGTGGA**GCA**ACTCAGCTAGCC
**RAD-K144A-R**	n/a	5′ GGCTAGCTGAGT**TGC**TCCACTACCG
**pRAD-TPX2/Y8A-F**	n/a	5' GGCGGCGGGCTTAAGAGC**GCT**AGC
**pRAD-TPX2/Y8A-R**	n/a	5' GCT**AGC**GCTCTTAAGCCCGCCGCC
**pRAD-TPX2/Y10A-F**	n/a	5' AGC**GCT**GATGCGCCGAGCGATTTTATTAAC
**pRAD-TPX2/Y10A-R**	n/a	5′ GTTAATAAAATCGCTCGGCGCATC**AGC**GCT
**pRAD-TPX2/D11A-F**	n/a	5′ CTATAGCTAT**GCT**GCGCCGAGCG
**pRAD-TPX2/D11A-R**	n/a	5' CGCTCGGCGC**AGC**ATAGCTATAG
**pRAD-TPX2/F16A-F**	n/a	5' GATGCGCCGAGCGAT**GCT**ATTAAC
**pRAD-TPX2/F16A-R**	n/a	5' GTTAAT**AGC**ATCGCTCGGCGCATC
**pRAD-TPX2/F19A-F**	n/a	5' ATTAAC**GCT**AGCAGCCTGGATGATG
**pRAD-TPX2/F19A-R**	n/a	5' CATCATCCAGGCTGCT**AGC**GTTAAT
**pRADATPX2/W34A-F**	n/a	5' ATTGATAGC**GCG**TTTGAAGAAAAAAAG
**pRAD-TPX2/W34A-R**	n/a	5' CTTTTTTTCTTCAAA**CGC**GCTATCAATG
**pRAD-TPX2/F35A-F**	n/a	5' GATAGCTGG**GCT**GAAGAAAAAGCG
**pRADA-TPX2/F35A-R**	n/a	5′ CGCTTTTTCTTC**AGC**CCAGCTATC
		**LIC Oligonucleotides**
**pRAD-LF1**	*Afl* II	5' *GGCGGCGGGCTTAA*GGTGGTTCTGGTGGTTCAGG 3′
**pRAD-LF2**	*Age* I	5' TGGTTCTGGTGGCAGCGGGGGCAGCACCGGTGGCGGGTCT
**pRAD-LF3**	*Bgl* II	5' GGCGGTTCAGGTGGTTCTGGTGGCAGCGGAGGGAGCAGAT
**pRAD-LF4**	*Bgl* II	5' CTGGCGGTGGGTCTGGTTAAGG
**pRAD-LR1**	n/a	5' CCCCGCTGCCACCAGAACCACCTGAACCACCAGAACCACCC
**pRAD-LR2**	*Age* I	5' CCAGAACCACCTGAACCGCCAGACCCGCCACCGGTGCTGC
**pRAD-LR3**	*Bgl* II	5' *CCGCCTCCCTTAA*CAGACCCACCGCCAGATCTGCTCCCTCCGCTGCC
**CK2β1**	*Afl* II	5' *GGCGGCGGGCTTAAG*CGCCTGTATGGCTTTAAAATTCATCCGATGGCGTATCAGCTGCAG*C*

**CK2β2**	*Afl* II	5' *CCGCCTCCCTTAAG*CTGCAGCTGATACGCCATCGGATGAATTTTAAAGCCATACAGGCG*C*
**TPX2-F1**	*Afl* II	5' *GGCGGCGGGCTTAAG*AGCTATAGCTATGATGCGCCGAGCG
**TPX2-F2**	n/a	5' ATTTTATTAACTTTAGCAGCCTGGATGATGAAGGCGATACCC
**TPX2-F3**	n/a	5' AGAACATTGATAGCTGGTTTGAAGAAAAAGCGAACCTGGAAAAC*C*
**TPX2-R3**	n/a	5' GGCTGCTAAAGTTAATAAAATCGCTCGGCGCATCATAGCTATAGCT*C*
**TPX2-R2**	n/a	5' CAAACCAGCTATCAATGTTCTGGGTATCGCCTTCATCATCCA
**TPX2-R1**	*Afl* II	5' *CCGCCTCCCTTAAG*GTTTTCCAGGTTCGCTTTTTCTT

Oligonucleotides used for the generation of the pRAD vectors, insertion of CK2β and TPX2 epitopes into pRAD vectors, cloning and alanine mutation of TPX2. Restriction enzyme recognition sites (when present) are underlined, mutation codon is highlighted in bold and LIC overhang in the peptide epitope encoding oligos are in italics.

Table S3. Proteins used in this study.

Protein construct	Expression plasmid	Purification step 1	Purification step 2
Aurora A$_{(125-403)}$ D274N	pHAT5	IMAC	GFC
Aurora A $_{(126-391)}$WT	pHAT4-LambdaPP	IMAC	GFC
CK2α $_{(2-329)}$	pBAT4	CEX	GFC
RAD-CK2β$_{(186-200)}$	pRAD1	CEX	GFC
cRAD-CK2β$_{(186-200)}$	pcRAD1	CEX	GFC
RAD-TPX2$_{(7-43)}$	pRAD1	CEX	GFC
cRAD-TPX2$_{(7-43)}$	pcRAD1	CEX	GFC
hchcRAD-TPX2$_{(7-43)}$	phahcRAD1	IMAC	GFC
hahcRAD-Y8A TPX2$_{(7-43)}$	phahcRAD1	IMAC	Buffer exchange
hahcRAD-Y10A TPX2$_{(7-43)}$	phahcRAD1	IMAC	Buffer exchange
hahcRAD-D11A TPX2$_{(7-43)}$	phahcRAD1	IMAC	Buffer exchange
hahcRAD-F16A TPX2$_{(7-43)}$	phahcRAD1	IMAC	Buffer exchange
hahcRAD-F19A TPX2$_{(7-43)}$	phahcRAD1	IMAC	Buffer exchange
hahcRAD-W34A TPX2$_{(7-43)}$	phahcRAD1	IMAC	Buffer exchange
hahcRAD-F35A TPX2$_{(7-43)}$	phahcRAD1	IMAC	Buffer exchange
TPX2$_{(7-43)}$	pET31b	IMAC	Reversed phase

Supplemental References:

1. Huet,S., Gorre,H., Perrocheau,A., Picot,J. and Cinier,M. (2015) Use of the Nanofitin Alternative Scaffold as a GFP-Ready Fusion Tag. *PLoS One*, **10**, e0142304.

2. Krehenbrink,M., Chami,M., Guilvout,I., Alzari,P.M., Pécorari,F. and Pugsley,A.P. (2008) Artificial Binding Proteins (Affitins) as Probes for Conformational Changes in Secretin PulD. *J. Mol. Biol.*, **383**, 1058–1068.

3. Behar,G., Bellinzoni,M., Maillasson,M., Paillard-Laurance,L., Alzari,P.M., He,X., Mouratou,B. and Pecorari,F. (2013) Tolerance of the archaeal Sac7d scaffold protein to alternative library designs: characterization of anti-immunoglobulin G Affitins. *Protein Eng. Des. Sel.*, **26**, 267–275.

4. Tiede,C., Tang,A.A.S., Deacon,S.E., Mandal,U., Nettleship,J.E., Owen,R.L., George,S.E., Harrison,D.J., Owens,R.J., Tomlinson,D.C., *et al.* (2014) Adhiron: a stable and versatile peptide display scaffold for molecular recognition applications. *Protein Eng. Des. Sel.*, **27**, 145–55.

5. Rawlings,A.E., Bramble,J.P., Tang,A.A.S., Somner,L.A., Monnington,A.E., Cooke,D.J., McPherson,M.J., Tomlinson,D.C. and Staniland,S.S. (2015) Phage display selected magnetite interacting Adhirons for shape controlled nanoparticle synthesis. *Chem. Sci.*, **6**, 5586–5594.

6. Škrlec,K., Štrukelj,B. and Berlec,A. (2015) Non-immunoglobulin scaffolds: a focus on their targets. *Trends Biotechnol.*, **33**, 408–418.

7. Hoffmann,T., Stadler,L.K.J., Busby,M., Song,Q., Buxton,A.T., Wagner,S.D., Davis,J.J. and Ferrigno,P.K. (2010) Structure-function studies of an engineered scaffold protein derived from stefin A. I: Development of the SQM variant. *Protein Eng. Des. Sel.*, **23**, 403–13.

8. Stadler,L.K.J., Tomlinson,D.C., Lee,T., Knowles,M. a and Ko Ferrigno,P. (2014) The use of a neutral peptide aptamer scaffold to anchor BH3 peptides constitutes a viable approach to studying their function. *Cell Death Dis.*, **5**, e1037.

9. Roberts,B.L., Markland,W., Ley, a C., Kent,R.B., White,D.W., Guterman,S.K. and Ladner,R.C. (1992) Directed evolution of a protein: selection of potent neutrophil elastase inhibitors displayed on M13 fusion phage. *Proc. Natl. Acad. Sci. U. S. A.*, **89**, 2429–2433.

10. Dennis,M.S. and Lazarus,R.A. (1994) Kunitz domain inhibitors of tissue factor-Factor VIIa. II. Potent and specific inhibitors by competitive phage selection. *J. Biol. Chem.*, **269**, 22137–22144.

11. Dennis,M.S. and Lazarus,R.A. (1994) Kunitz domain inhibitors of tissue factor-Factor VIIa. I. Potent inhibitors selected from libraries by phage display. *J. Biol. Chem.*, **269**, 22129–22136.

12. Stoop, a A. and Craik,C.S. (2003) Engineering of a macromolecular scaffold to develop specific protease inhibitors. *Nat. Biotechnol.*, **21**, 1063–8.

13. Lehmann,A. (2008) Ecallantide (DX-88), a plasma kallikrein inhibitor for the treatment of hereditary angioedema and the prevention of blood loss in on-pump cardiothoracic surgery. *Expert Opin. Biol. Ther.*, **8**, 1187–1199.

14. Nilverbrant,J., Alm,T., Hober,S. and Lofblom,J. (2011) Engineering Bispecificity into a single Albumin-binding Domain. *PLoS One*, **6**, 1–13.

15. Nilverbrant,J. (2012) An albumin-binding domain as a scaffold for bispecific affinity proteins An albumin-binding domain as a scaffold for bispecific affinity proteins.

16. Marečková,L., Petroková,H., Osička,R., Kuchař,M. and Malý,P. (2015) Novel binders derived from an albumin-binding domain scaffold targeting human prostate secretory protein 94 (PSP94). *Protein Cell*, **6**, 774–779.

17. Miao,Z., Levi,J. and Cheng,Z. (2011) Protein scaffold-based molecular probes for cancer molecular imaging. *Amino Acids*, **41**, 1037–47.

18. Jonsson,A. (2009) Development of molecular recognition by rational and combinatorial engineering.

19. Feldwisch,J., Tolmachev,V., Lendel,C., Herne,N., Sjöberg,A., Larsson,B., Rosik,D., Lindqvist,E., Fant,G., Höidén-Guthenberg,I., *et al.* (2010) Design of an Optimized Scaffold for Affibody Molecules. *J. Mol. Biol.*, **398**, 232–247.

20. Löfblom,J., Feldwisch,J., Tolmachev,V., Carlsson,J., Ståhl,S. and Frejd,F.Y. (2010)

Affibody molecules: Engineered proteins for therapeutic, diagnostic and biotechnological applications. *FEBS Lett.*, **584**, 2670–2680.

21. Lipovšek,D. (2011) Adnectins: engineered target-binding protein therapeutics. *Protein Eng. Des. Sel.*, **24**, 3–9.

22. Ramamurthy,V., Krystek,S.R., Bush,A., Wei,A., Emanuel,S.L., Das Gupta,R., Janjua,A., Cheng,L., Murdock,M., Abramczyk,B., *et al.* (2012) Structures of adnectin/protein complexes reveal an expanded binding footprint. *Structure*, **20**, 259–269.

23. Koide,A., Wojcik,J., Gilbreth,R.N., Hoey,R.J. and Koide,S. (2012) Teaching an old scaffold new tricks: Monobodies constructed using alternative surfaces of the FN3 scaffold. *J. Mol. Biol.*, **415**, 393–405.

24. Parker,M.W. and Feil,S.C. (2005) Pore-forming protein toxins: From structure to function. *Prog. Biophys. Mol. Biol.*, **88**, 91–142.

25. Cappuccilli,G., Crea,R., Shen,R., Hokanson,C.A., Kirk,P.B. and Liston,D.R. (2013) Universal Fibronectin type III binding-domain libraries. Google Patents.

26. Diem,M.D., Hyun,L., Yi,F., Hippensteel,R., Kuhar,E., Lowenstein,C., Swift,E.J., O'Neil,K.T., Jacobs,S.A. and Marx,J. (2014) Selection of high-affinity Centyrin FN3 domains from a simple library diversified at a combination of strand and loop positions. *Protein Eng. Des. Sel.*, **27**, 419–429.

27. Moody,I.S., Verde,S.C., Overstreet,C.M., Edward Robinson,W. and Weiss,G. a (2012) In vitro evolution of an HIV integrase binding protein from a library of C-terminal domain γS-crystallin variants. *Bioorg. Med. Chem. Lett.*, **22**, 5584–9.

28. Ebersbach,H., Fiedler,E., Scheuermann,T., Fiedler,M., Stubbs,M.T., Reimann,C., Proetzel,G., Rudolph,R. and Fiedler,U. (2007) Affilin–Novel Binding Molecules Based on Human γ-B-Crystallin, an All β-Sheet Protein. *J. Mol. Biol.*, **372**, 172–185.

29. Mirecka,E. a., Hey,T., Fiedler,U., Rudolph,R. and Hatzfeld,M. (2009) Affilin Molecules Selected against the Human Papillomavirus E7 Protein Inhibit the Proliferation of Target Cells. *J. Mol. Biol.*, **390**, 710–721.

30. Hoffmann,A., Kovermann,M., Lilie,H., Fiedler,M., Balbach,J., Rudolph,R. and Pfeifer,S. (2012) New binding mode to TNF-alpha revealed by ubiquitin-based artificial binding protein. *PLoS One*, **7**, 2–11.

31. Lorey,S., Fiedler,E., Kunert,A., Nerkamp,J., Lange,C., Fiedler,M., Bosse-Doenecke,E., Meysing,M., Gloser,M., Rundfeldt,C., *et al.* (2014) Novel Ubiquitin-Derived High Affinity Binding Proteins with Tumor Targeting Properties. *J. Biol. Chem.*, **289**, 8493–8507.

32. Richter, a, Eggenstein,E. and Skerra, a (2014) Anticalins: Exploiting a non-Ig scaffold with hypervariable loops for the engineering of binding proteins. *FEBS Lett.*, **588**, 213–8.

33. Schlehuber,S. and Skerra,A. (2002) Tuning ligand affinity, specificity, and folding stability of an engineered lipocalin variant - A so-called 'anticalin' - Using a molecular random approach. *Biophys. Chem.*, **96**, 213–228.

34. Filimonov,V. V, Azuaga, a I., Viguera, a R., Serrano,L. and Mateo,P.L. (1999) A thermodynamic analysis of a family of small globular proteins: SH3 domains. *Biophys. Chem.*, **77**, 195–208.

35. Grabulovski,D., Kaspar,M. and Neri,D. (2007) A novel, non-immunogenic Fyn SH3-

derived binding protein with tumor vascular targeting properties. *J. Biol. Chem.*, **282**, 3196–3204.

36. Schlatter,D., Brack,S., Banner,D.W., Batey,S., Benz,J., Bertschinger,J., Huber,W., Joseph,C., Rufer,A.C., Van Der Klooster,A., *et al.* (2012) Generation, characterization and structural data of chymase binding proteins based on the human Fyn kinase SH3 domain. *MAbs*, **4**, 497–508.

37. Steemson,J.D., Baake,M., Rakonjac,J., Arcus,V.L. and Liddament,M.T. (2014) Tracking Molecular Recognition at the Atomic Level with a New Protein Scaffold Based on the OB-Fold. *PLoS One*, **9**, e86050.

38. Silverman,A.P., Levin,A.M., Lahti,J.L. and Cochran,J.R. (2009) Engineered Cystine-Knot Peptides that Bind avb3 Integrin with Antibody-Like Affinities. *J. Mol. Biol.*, **385**, 1064–1075.

39. Kimura,R.H., Levin,A.M., Cochran,F. V. and Cochran,J.R. (2009) Engineered cystine knot peptides that bind avb3, avb5, and a5b1 integrins with low-nanomolar affinity. *Proteins Struct. Funct. Bioinforma.*, **77**, 359–369.

40. Moore,S.J. and Cochran,J.R. (2012) Engineering knottins as novel binding agents 1st ed. Elsevier Inc.

41. Desmet,J., Verstraete,K., Bloch,Y., Lorent,E., Wen,Y., Devreese,B., Vandenbroucke,K., Loverix,S., Hettmann,T., Deroo,S., *et al.* (2014) Structural basis of IL-23 antagonism by an Alphabody protein scaffold. *Nat. Commun.*, **5**, 5237.
